# Microbial diversity of vermicompost bacteria that exhibit useful agricultural traits and waste management potential

**DOI:** 10.1186/2193-1801-1-26

**Published:** 2012-10-04

**Authors:** Jayakumar Pathma, Natarajan Sakthivel

**Affiliations:** Department of Biotechnology School of Life Sciences, Pondicherry University, Kalapet, Puducherry, 605014 India

**Keywords:** Vermicompost, Earthworms, Beneficial bacteria, Organic waste management, Pathogen suppression, Plant-growth promotion, Biofertilizer

## Abstract

Vermicomposting is a non-thermophilic, boioxidative process that involves earthworms and associated microbes. This biological organic waste decomposition process yields the biofertilizer namely the vermicompost. Vermicompost is a finely divided, peat like material with high porosity, good aeration, drainage, water holding capacity, microbial activity, excellent nutrient status and buffering capacity thereby resulting the required physiochemical characters congenial for soil fertility and plant growth. Vermicompost enhances soil biodiversity by promoting the beneficial microbes which inturn enhances plant growth directly by production of plant growth-regulating hormones and enzymes and indirectly by controlling plant pathogens, nematodes and other pests, thereby enhancing plant health and minimizing the yield loss. Due to its innate biological, biochemical and physiochemical properties, vermicompost may be used to promote sustainable agriculture and also for the safe management of agricultural, industrial, domestic and hospital wastes which may otherwise pose serious threat to life and environment.

## Introduction

Soil, is the soul of infinite life that promotes diverse microflora. Soil bacteria viz., *Bacillus, Pseudomonas* and *Streptomyces* etc., are prolific producers of secondary metabolites which act against numerous co-existing phytopathogeic fungi and human pathogenic bacteria (Pathma et al. 2011b). Earthworms are popularly known as the “farmer’s friend” or “nature’s plowman”. Earthworm influences microbial community, physical and chemical properties of soil. They breakdown large soil particles and leaf litter and thereby increase the availability of organic matter for microbial degradation and transforms organic wastes into valuable vermicomposts by grinding and digesting them with the help of aerobic and anaerobic microbes (Maboeta and Van Rensburg 2003). Earthworms activity is found to enhance the beneficial microflora and suppress harmful pathogenic microbes. Soil wormcasts are rich source of micro and macro-nutrients, and microbial enzymes (Lavelle and Martin 1992). Vermicomposting is an efficient nutrient recycling process that involves harnessing earthworms as versatile natural bioreactors for organic matter decomposition. Due to richness in nutrient availability and microbial activity vermicomposts increase soil fertility, enhance plant growth and suppress the population of plant pathogens and pests. This review paper describes the bacterial biodiversity and nutrient status of vermicomposts and their importance in agriculture and waste management.

### Earthworms

Earthworms are capable of transforming garbage into ‘gold’. Charles Darwin described earthworms as the ‘unheralded soldiers of mankind’, and Aristotle called them as the ‘intestine of earth’, as they could digest a wide variety of organic materials (Darwin and Seward 1903; Martin 1976). Soil volume, microflora and fauna influenced by earthworms have been termed as "drilosphere" and the soil volume includes the external structures produced by earthworms such as surface and below ground casts, burrows, middens, diapause chambers as well as the earthworms body surface and internal gut associated structures in contact with the soil (Lavelle et al. 1989; Brown et al. 2000). Earthworms play an essential role in carbon turnover, soil formation, participates in cellulose degradation and humus accumulation. Earthworm activity profoundly affects the physical, chemical and biological properties of soil. Earthworms are voracious feeders of organic wastes and they utilize only a small portion of these wastes for their growth and excrete a large proportion of wastes consumed in a half digested form (Edwards and Lofty 1977; Kale and Bano 1986; Jambhekar 1992). Earthworms intestine contains a wide range of microorganisms, enzymes and hormones which aid in rapid decomposition of half-digested material transforming them into vermicompost in a short time (neary 4–8 weeks) (Ghosh et al. 1999; Nagavallemma et al. 2004) compared to traditional composting process which takes the advantage of microbes alone and thereby requires a prolonged period (nearly 20 weeks) for compost production (Bernal et al. 1998; Sánchez-Monedero et al. 2001). As the organic matter passes through the gizzard of the earthworm it is grounded into a fine powder after which the digestive enzymes, microorganisms and other fermenting substances act on them further aiding their breakdown within the gut, and finally passes out in the form of “casts” which are later acted upon by earthworm gut associated microbes converting them into mature product, the “vermicomposts” (Dominguez and Edwards 2004).

Earthworms, grouped under phylum annelida are long, narrow, cylindrical, bilaterally symmetrical, segmented soil dwelling invertebrates with a glistening dark brown body covered with delicate cuticle. They are hermaphrodites and weigh over 1,400–1,500 mg after 8–10 weeks. Their body contains 65% protein (70–80% high quality ‘lysine rich protein’ on a dry weight basis), 14% fats, 14% carbohydrates, and 3% ash. Their life span varies between 3–7 years depending upon the species and ecological situation. The gut of earthworm is a straight tube starting from mouth followed by a muscular pharynx, oesophagus, thin walled crop, muscular gizzard, foregut, midgut, hindgut, associated digestive glands, and ending with anus. The gut consisted of mucus containing protein and polysaccharides, organic and mineral matter, amino acids and microbial symbionts viz., bacteria, protozoa and microfungi. The increased organic carbon, total organic carbon and nitrogen and moisture content in the earthworm gut provide an optimal environment for the activation of dormant microbes and germination of endospores etc. A wide array of digestive enzymes such as amylase, cellulase, protease, lipase, chitinase and urease were reported from earthworm's alimentary canal. The gut microbes were found to be responsible for the cellulase and mannose activities (Munnoli et al. 2010). Earthworms comminutes the substrate, thereby increases the surface area for microbial degradation constituting to the active phase of vermicomposting. As this crushed organic matter passes through the gut it get mixed up with the gut associated microbes and the digestive enzymes and finally leaves the gut in partially digested form as “casts” after which the microbes takes up the process of decomposition contributing to the maturation phase (Lazcano et al. 2008).

Association of earthworms with microbes is found to be complex. Certain groups of microbes were found to be a part of earthworm’s diet which is evidenced by the destruction of certain microbes as they pass through the earthworms digestive system. Few yeasts, protozoa and certain groups of fungi such as *Fusarium oxysporum, Alternaria solani*, and microfungi were digested by the earthworms, *Drawida calebi, Lumbricus terrestris* and *Eisenia foetida*. *Bacillus cereus* var *mycoides* were reported to decrease during gut passage while *Escherichia coli* and *Serratia marcessens* were completely eliminated during passage through earthworm gut (Edwards and Fletcher, 1988).

Earthworms are classified into epigeic, anecic and endogeic species based on definite ecological and trophic functions (Brown 1995; Bhatnagar and Palta 1996) (Table 1). Epigeic earthworms are smaller in size, with uniformly pigmented body, short life cycle, high reproduction rate and regeneration. They dwell in superficial soil surface within litters, feeds on the surface litter and mineralize them. They are phytophagous and rarely ingest soil. They contain an active gizzard which aids in rapid conversion of organic matter into vermicomposts. In addition epigeic earthworms are efficient bio-degraders and nutrient releasers, tolerant to disturbances, aids in litter comminution and early decomposition and hence can be efficiently used for vermicomposting. Epigeic earthworms includes *Eisenia foetida, Lumbricus rubellus, L. castaneus, L. festivus, Eiseniella tetraedra, Bimastus minusculus, B. eiseni, Dendrodrilus rubidus, Dendrobaena veneta, D. octaedra.* Endogeics earthworms are small to large sized worms, with weakly pigmented body, life cycle of medium duration, moderately tolerant to disturbance, forms extensive horizontal burrows and they are geophagous feeding on particulate organic matter and soil. They bring about pronounced changes in soil physical structure and can efficiently utilize energy from poor soils, hence can be used for soil improvements. Endogeics include *Aporrectodea caliginosa*, *A. trapezoides, A. rosea, Millsonia anomala, Octolasion cyaneum, O. lacteum, Pontoscolex corethrurus, Allolobophora chlorotica* and *Aminthas* sp*.* They are further classified into polyhumic endogeic which are small sized, rich soil feeding earthworms, dwelling in top soil (A1); mesohumic endogeic which are medium sized worms, dwelling in A and B horizon, feeding on bulk (A1) soil; and oligohumic endogeic which are very large worms, dwelling in B and C horizons, feeding on poor, deep soil. Aneceics are larger, dorsally pigmented worms, with low reproductive rate, sensitive to disturbance, nocturnal, phytogeophagous, bury the surface litter, forms middens and extensive, deep, permanent vertical burrows, and live in them. Formation of vertical burrows affects airwater relationship and movement from deep layers to surface helps in efficient mixing of nutrients. *Lumbricus terrestris, L. polyphemus* and *Aporrectodea longa* are examples of aneceics earthworms (Kooch and Jalilvand 2008). Epigeics and aneceics are harnessed largely for vermicomposting (Asha et al. 2008). Epigeics namely *Eisenia foetida* (Hartenstein et al. 1979), *Eudrilus eugeniae* (Kale and Bano 1988), *Perionyx excavatus* (Sinha et al. 2002; Suthar and Singh 2008) and *Eisenia anderi* (Munnoli et al. 2010) have been used in converting organic wastes into vermicompost. These surface dwellers capable of working on litter layers converting them into manure are of no significant value in modifying the soil structure. In contrast, anecics such as *Lampito mauritii* are efficient creators of an effective drilosphere as well as excellent compost producers (Ismail 1997). Earthworms thus act as natural bio-reactors, altering the nature of the organic waste by fragmenting them.

**Table 1:** **Ecological categories and niches of earthworms and their characteristic features and beneficial traits**

Species	Ecological category	Ecological niche	Characteristic features	Beneficial trait
*Eisenia foetida,*	Epigeics	Superficial soil layers, leaf litter, compost	Smaller in size, body uniformly pigmented, active gizzard, short life cycle, high reproduction rate and regeneration, tolerant to disturbance, phytophagous	Efficient bio-degraders and nutrient releasers, efficient compost producers, aids in litter comminution and early decomposition
*Lumbricus rubellus,*				
*L. castaneus,*				
*L. festivus,*				
*Eiseniella tetraedra,*				
*Bimastus minusculus,*				
*B. eiseni,*				
*Dendrodrilus rubidus,*				
*Dendrobaena veneta,*				
*D. octaedra*				
*Aporrectodea caliginosa*,	Endogeics	Topsoil or subsoil	Small to large sized worms, weakly pigmented, life cycle of medium duration, moderately tolerant to disturbance, geophagous	Brings about pronounced changes in soil physical structure, can efficiently utilize energy from poor soils hence can be used for soil improvements
*A. trapezoides,*				
*A. rosea,*				
*Millsonia anomala,*				
*Octolasion cyaneum,*	Polyhumic endogeic	Top soil (A_1_)	Small size, unpigmented, forms horizontal burrows, rich soil feeder	
*O. lacteum,*				
*Pontoscolex corethrurus,*	Mesohumic endogeic	A and B horizon	Medium size, unpigmented, forms extensive horizontal burrows, bulk (A_1_) soil feeder	
*Allolobophora chlorotica,*				
*Aminthas sp.*	Oligohumic endogeic	B and C horizon	Very large in size, unpigmented, forms extensive horizontal burrows, feeds on poor, deep soils	
*L. terrestris,*	Anecics	Permanent deep burrows in soil	Large in size, dorsally pigmented, forms extensive, deep, vertical permanent burrows, low reproductive rate, sensitive to disturbance, phytogeophagous, nocturnal	Forms vertical burrows affecting air-water relationship and movement from deep layers to surface helps in efficient mixing of nutrients
*L. polyphemus,*				
*A. longa*				

Earthworm activity engineers the soil by forming extensive burrows which loosen the soil and makes it porous. These pores improve aeration, water absorption, drainage and easy root penetration. Soil aggregates formed by earthworms and associated microbes, in the casts and burrow walls play an indispensible role in soil air ecosystem. These aggregates are mineral granules bonded in a way to resist erosion and to avoid soil compaction both in wet and dry condition. Earthworms speed up soil reclamation and make them productive by restoring beneficial microflora (Nakamura 1996). Thus degraded unproductive soils and land degraded by mining could be engineered physically, chemically and biologically and made productive by earthworms. Hence earthworms are termed as ecosystem engineers (Brown et al. 2000; Munnoli et al. 2010).

### Vermicomposting

Vermicomposting is a non-thermophilic biological oxidation process in which organic material are converted into vermicompost which is a peat like material, exhibiting high porosity, aeration, drainage, water holding capacity and rich microbial activities (Edwards 1998; Atiyeh et al. 2000b; Arancon et al. 2004a), through the interactions between earthworms and associated microbes. Vermiculture is a cost-effective tool for environmentally sound waste management (Banu et al. 2001; Asha et al. 2008). Earthworms are the crucial drivers of the process, as they aerate, condition and fragment the substrate and thereby drastically alter the microbial activity and their biodegradation potential (Fracchia et al. 2006; Lazcano et al. 2008). Several enzymes, intestinal mucus and antibiotics in earthworm’s intestinal tract play an important role in the breakdown of organic macromolecules. Biodegradable organic wastes such as crop residues, municipal, hospital and industrial wastes pose major problems in disposal and treatment. Release of unprocessed animal manures into agricultural fields contaminates ground water causing public health risk. Vermicomposting is the best alternative to conventional composting and differs from it in several ways (Gandhi et al. 1997). Vermicomposting hastens the decomposition process by 2–5 times, thereby quickens the conversion of wastes into valuable biofertilizer and produces much more homogenous materials compared to thermophilic composting (Bhatnagar and Palta 1996; Atiyeh et al. 2000a). Distinct differences exist between the microbial communities found in vermicomposts and composts and hence the nature of the microbial processes is quite different in vermicomposting and composting (Subler et al. 1998). The active phase of composting is the thermophilic stage characterized by thermophilic bacterial community where intensive decomposition takes place followed by a mesophilic maturation phase (Lazcano et al. 2008; Vivas et al. 2009). Vermicomposting is a mesophilic process characterized by mesophilic bacteria and fungi (Benitez et al. 1999). Vermicomposting comprises of an active stage during which earthworms and associated microbes jointly process the substrate and the maturation phase that involves the action of associated microbes and occurs once the worm’s moves to the fresher layers of undigested waste or when the product is removed from the vermireacter. The duration of the active phase depends on the species and density of the earthworms involved (Ndegwa et al. 2000; Lazcano et al. 2008; Aira et al. 2011). A wide range of oganic wastes viz., horticultural residues from processed potatoes (Edwards 1988); mushroom wastes (Edwards 1988; Tajbakhsh et al. 2008); horse wastes (Hartenstein et al. 1979; Edwards et al. 1998); pig wastes (Chan and Griffiths 1988; Reeh 1992); brewery wastes (Butt 1993); sericulture wastes (Gunathilagraj and Ravignanam 1996); municipal sewage sludge (Mitchell et al. 1980; Dominguez et al. 2000); agricultural residues (Bansal and Kapoor 2000); weeds (Gajalakshmi et al. 2001); cattle dung (Gunadi et al. 2002); industrial refuse such as paper wastes (Butt 1993; Elvira et al. 1995; Gajalakshmi et al. 2002); sludge from paper mills and dairy plants (Elvira et al. 1997; Banu et al. 2001); domestic kitchen wastes (Sinha et al. 2002); urban residues and animal wastes (Edwards et al. 1985; Edwards 1988) can be vermicomposted (Sharma et al. 2005).

Effects of vermicomposting on pH, electrical conductivity (EC), C:N ratio and other nutrients have been documented. Earthworm activity reduced pH and C:N ratio in manure (Gandhi et al. 1997; Atiyeh et al. 2000b). Chemical analysis showed vermicompost had a lower pH, EC, organic carbon (OC) (Nardi et al. 1983; Albanell et al. 1988; Mitchell 1997), C:N ratio (Riffaldi and Levi-Minzi 1983; Albanell et al. 1988), nitrogen and potassium and higher amounts of total phosphorous and micronutrients compared to the parent material (Hashemimajd et al. 2004). Slightly decreased pH values of vermicompost compared to traditional compost might be attributed due to mineralization of N and P, microbial decomposition of organic materials into intermediate organic acids, fulvic acids, humic acids (Lazcano et al. 2008; Albanell et al. 1988; Chan and Griffiths 1988; Subler et al. 1998) and concomitant production of CO_2_ (Elvira et al. 1998; Garg et al. 2006). Vermicomposting of paper mill and dairy sludge resulted in 1.2–1.7 fold loss of organic carbon as CO_2_ (Elvira et al. 1998). In contrast to the parent material used, vermicomposts contain higher humic acid substances (Albanell et al. 1988). Humic acid substances occur naturally in mature animal manure, sewage sludge or paper-mill sludge, but vermicomposting drastically increases the rate of production and their amount from 40–60 percent compared to traditional composting. The enhancement in humification processes is by fragmentation and size reduction of organic matter, increased microbial activity within earthworm intestine and soil aeration by earthworm feeding and movement (Dominguez and Edwards, 2004). EC indicates the salinity of the organic amendment. Minor production of soluble metabolites such as ammonium and precipitation of dissolved salts during vermicomposting lead to lower EC values. Compared to the parent material used, vermicomposts contain less soluble salts and greater cation exchange capacity (Holtzclaw and Sposito 1979; Albanell et al. 1988). C:N ratio is an indicator of the degree of decomposition. During the process of biooxidation, CO_2_ and N is lost and loss of N takes place at a comparatively lower rate. Comparison of compost and vermicompost showed that vermicompost had significantly less C:N ratios as they underwent intense decomposition (Lazcano et al. 2008).

Vermicomposting of cow manure using earthworm species *E. andrei* (Atiyeh et al. 2000b) and *E*. *foetida* (Hand et al. 1988) favored nitrification, resulting in the rapid conversion of ammonium-nitrogen to nitrate-nitrogen. Vermicomposting increased the concentration of nitrate-nitrogen to 28 fold after 17 weeks, while in conventional compost there was only 3-fold increase (Subler et al. 1998; Atiyeh et al. 2000a). Increase in ash concentration during vermicomposting suggests that vermicomposting accelerates the rate of mineralization (Albanell et al. 1988). Mineralization is the process in which the chemical compounds in the organic matter decompose or oxidise into forms that could be easily assimilated by the plants. Increase in ash content increases the rate of mineralization. Ash is an alkaline substance which hinders the formation of H_2_S as well as improves the availibility of O_2_ and thereby renders composts odorless. Thus vermicomposting increases the ash content and accelerates the rate of mineralization which is essential to make nutrients available to plants. The observed increase of total phosphorous (TP) in vermicompost is probably due to mineralization and mobilization of phosphorus resulting from the enhanced phosphatase activity by microorganisms in the gut epithelium of the earthworms (Zhang et al. 2000; Garg et al. 2006). Vermicomposts showed a significant increase in exchangeable Ca^2+^, Mg^2+^ and K^+^ compared to fresh sludge indicating the conversion of nutrients to plant-available forms during passage through the earthworm gut (Garg et al. 2006; Yasir et al. 2009a). Vermicomposts contain higher nutrient concentrations, but less likely to produce salinity, than composts. Additionally, vermicomposts possess outstanding biological properties and have microbial populations significantly larger and more diverse compared to conventional composts (Edwards 1998). Soil supplemented with vermicompost showed better plant growth compared to soil treated with inorganic fertilizers or cattle manure (Kalembasa 1996; Subler et al. 1998).

### Diversity of bacteria associated with earthworms

Earthworm’s ability to increase plant nutrient availability is likely to be dependent on the activity of earthworm gut microflora. Earthworms indirectly influence the dynamics of soil chemical processes, by comminuting the litter and affecting the activity of the soil micro-flora (Petersen and Luxton 1982; Lee 1985; Edwards and Bohlen 1996). Interactions between earthworms and microorganisms seem to be complex. Earthworms ingest plant growth-promoting rhizospheric bacteria such as *Pseudomonas, Rhizobium, Bacillus, Azosprillium, Azotobacter,* etc. along with rhizospheric soil, and they might get activated or increased due to the ideal micro-environment of the gut. Therefore earthworm activity increases the population of plant growth-promoting rhizobacteria (PGPR) (Sinha et al. 2010). This specific group of bacteria stimulates plant growth directly by solubilization of nutrients (Ayyadurai et al. 2007; Ravindra et al. 2008), production of growth hormone, 1-aminocyclopropane-1-carboxylate (ACC) deaminase (Correa et al. 2004), nitrogen fixation (Han et al. 2005), and indirectly by suppressing fungal pathogens. Antibiotics, fluorescent pigments, siderophores and fungal cell-wall degrading enzymes namely chitinases and glucanases (Han et al. 2005; Sunish et al. 2005; Ravindra et al. 2008; Jha et al. 2009; Pathma et al. 2010; Pathma et al. 2011a, b) produced by bacteria mediate the fungal growth-suppression. Earthworms are reported to have association with such free living soil bacteria and constitute the drilosphere (Ismail 1995). Earthworm microbes mineralize the organic matter and also facilitate the chelation of metal ions (Pizl and Novokova 1993; Canellas et al. 2002). Gut of earthworms *L. terrestris, Allolobophora caliginosa* and *Allolobophora terrestris* were reported to contain higher number of aerobes compared to soil (Parle 1963). Earthworms increased the number of microorganisms in soi1 as much as five times (Edwards and Lofty 1977) and the number of bacteria and ‘actinomycetes’ contained in the ingested material increased upto 1,000 fold while passing through their gut (Edwards and Fletcher 1988). Similar increase was observed in plate counts of total bacteria, proteolytic bacteria and actinomycetes by passage through earthworms gut (Parle 1963; Daniel and Anderson 1992; Pedersen and Hendriksen 1993; Devliegher and Verstraete 1995). Similarly microbial biomass either decreased (Bohlen and Edwards 1995; Devliegher and Verstraete 1995), or increased (Scheu 1992) or remained unchanged (Daniel and Anderson 1992) after passage through the earthworm gut. An oxalate-degrading bacterium *Pseudomonas oxalaticus* was isolated from intestine of *Pheretima* species (Khambata and Bhat 1953) and an actinomycete *Streptomyces lipmanii* was identified in the gut of *Eisenia lucens* (Contreras 1980). Scanning electron micrographs provided evidence for endogenous microflora in guts of earthworms, *L. terrestris* and *Octolasion cyaneum* (Jolly et al. 1993). Gut of *E. foetida* contained various anaerobic N_2_-fixing bacteria such as *Clostridium butyricum, C. beijerinckii* and *C. paraputrificum* (Citernesi et al. 1977). Alimentary canal of *Lumbricus rubellus* and *Octolasium lacteum* were found to contain more numbers of aerobes and anerobes (Karsten and Drake 1995) and culturable denitrifiers (Karsten and Drake 1997). List of vermicompost bacteria and their beneficial traits is presented in Table 2.

**Table 2:** **Biodiversity of vermicompost bacteria and their beneficial traits**

Vermicompost earthworm	Names of bacteria	Beneficial traits	References
*Pheretima* sp.	*Pseudomonas oxalaticus*	Oxalate degradation	Khambata and Bhat, 1953
Unspecified	*Rhizobium trifolii*	Nitrogen fixation and growth of leguminous plants	Buckalew et al. 1982
*Lumbricus rubellus*	*R. japonicum, P. putida*	Plant growth promotion	Madsen and Alexander 1982
*L. terrestris*	*Bradyrhizobium japonicum*	Improved distribution of nodules on soybean roots	Rouelle, 1983
*Aporrectodea trapezoids,*	*P. corrugata 2*14OR	Suppress *Gaeumannomyces graminis* var. *Tritd* in wheat	Doube et al. 1994
*A. rosea*			
*A. trapezoids,*	*R. meliloti* L5-30R	Increased root nodulation and nitrogen fixation in legumes	Stephens et al. 1994b
*Microscolex dubius*			
*Eisenia foetida*	*Bacillus* spp.*, B. megaterium,*	Antimicrobial activity against *Enterococcus faecalis*DSM 2570, *Staphylococcus aureus* DSM 1104	Vaz-Moreira et al. 2008
	*B. pumilus, B. subtilis*		
*L. terrestris*	Fluorescent pseudomonads,	Suppress *Fusarium oxysporum* f. sp. *asparagi* and *F. proliferatum* in asparagus, *Verticillium dahlia* in eggplant and *F. oxysporum* f. sp*. lycopersici* Race 1 in tomato	Elmer, 2009
	Filamentous actinomycetes		
*Eudrilus* sp.	Free-living N_2_ fixers,	Plant growth promotion by nitrification, phosphate solubilisation and plant disease suppression	Gopal et al. 2009
	*Azospirillum*, *Azotobacter,*		
	Autotrophic *Nitrosomonas*,		
	*Nitrobacter*, Ammonifying		
	bacteria, Phosphate solubilizers,		
	Fluorescent pseudomonads		
*E. foetida*	Proteobacteria, Bacteroidetes,	Antifungal activity against *Colletotrichum coccodes, R. solani, P. ultimum, P. capsici* and *F. moliniforme*	Yasir et al. 2009a
	Verrucomicrobia, Actinobacteria,		
	Firmicutes		
Unspecified	*Eiseniicola composti* YC06271^T^	Antagonistic activity against *F*. *moniliforme*	Yasir et al. 2009b

Earthworms harbor ‘nitrogen-fixing’ and ‘decomposer microbes’ in their gut and excrete them along with nutrients in their excreta (Singleton et al. 2003). Earthworms stimulate and accelerate microbial activities by increasing the population of soil microorganisms (Binet et al. 1998), microbial numbers and biomass (Edwards and Bohlen 1996), by improving aeration through burrowing actions. Vermicomposting modified the original microbial community of the waste in a diverse way. Actinobacteria and Gammaproteobacteria were abundant in vermicompost, while conventional compost contained more Alphaproteobacteria and Bacteriodetes, the bacterial phylogenetic groups typical of non-cured compost (Vivas et al. 2009). Total bacterial counts exceeded 10^- 10^/ g of vermicompost and it included nitrobacter, azotobacter, rhizobium, phosphate solubilizers and actinomycetes (Suhane 2007). Molecular and culture-dependent analyses of bacterial community of vermicompost showed the presence of α-Proteobacteria, β-Proteobacteria, γ-Proteobacteria, Actinobacteria, Planctomycetes, Firmicutes and Bacteroidetes (Yasir et al. 2009a). Several findings showed considerable increase in total viable counts of actinomycetes and bacteria in the worm treated compost (Parthasarathi and Ranganathan 1998; Haritha Devi et al. 2009). The increase of microbial population may be due to the congenial condition for the growth of microbes within the digestive tract of earthworm and by the ingestion of nutrient rich organic wastes which provide energy and also act as a substrate for the growth of microorganisms (Tiwari et al. 1989). The differences in microbial species, numbers and activity between the earthworm alimentary canal or burrow and bulk soil indirectly support the hypothesis that the bacterial community structures of these habitats are different from those of the soil. Specific phylogenetic groups of bacteria such as *Aeromonas hydrophila* in *E. foetida* (Toyota and Kimura 2000), fluorescent pseudomonads in *L. terrestris* (Devliegher and Verstraete 1997), and *Actinobacteria* in *L. rubellus* (Kristufek et al. 1993) have been found in higher numbers in earthworm guts, casts, or burrows.

Enzymatic activity characterization and quantification has a direct correlation with type and population of microbes and reflects the dynamics of the composting process in terms of the decomposition of organic matter and nitrogen transformations and provide information about the maturity of the compost (Tiquia 2005). Wormcasts contain higher activities of cellulase, amylase, invertase, protease, peroxidase, urease, phosphatase and dehydrogenase (Sharpley and Syers 1976; Edwards and Bohlen 1996). Dehydrogenase is an intracellular enzyme related to the oxidative phosphorylation process (Trevors 1984) and is an indicator of microbial activity in soil and other biological ecosystems (Garcia et al. 1997). The maximum enzyme activities (cellulase, amylase, invertase, protease and urease) were observed during 21–35 days in vermicomposting and on 42–49 days in conventional composting. Also, microbial numbers and their extracellular enzyme profiles were more abundant in vermicompost produced from fruitpulp, vegetable waste, groundnut husk and cowdung compared to the normal compost of the same parental origin (Haritha Devi et al. 2009). *Pseudomonas, Paenibacillus, Azoarcus, Burkholderia, Spiroplasm, Acaligenes,* and *Acidobacterium*, the potential degraders of several categories of organics are seen associated with the earthworm’s intestine and vermicasts (Singleton et al. 2003). Firmicutes viz., *Bacillus benzoevorans, B. cereus*, *B. licheniformis*, *B. megaterium*, *B. pumilus, B. subtilis, B. macroides*; Actinobacteria namely *Cellulosimicrobium cellulans, Microbacterium* spp., *M. oxydans*; Proteobacteria such as *Pseudomonas* spp., *P. libaniensis*; ungrouped genotypes *Sphingomonas* sp., *Kocuria palustris* and yeasts namely *Geotrichum* spp. and *Williopsis californica* were reported from vermicomposts (Vaz-Moreira et al. 2008). Pinel et al*.* (2008) reported the presence of a novel nephridial symbiont, *Verminephrobacter eiseniae* from *E. foetida. Ochrobactrum* sp., *Massilia* sp., *Leifsonia* sp. and bacteria belonging to families Aeromonadaceae, Comamonadaceae, Enterobacteriaceae, Flavobacteriaceae, Moraxellaceae, Pseudomonadaceae, Sphingobacteriaceae, Actinobacteria and Microbacteriaceae were reported to occur in earthworms alimentary canal (Byzov et al. 2009). The microbial flora of earthworm gut and cast are potentially active and can digest a wide range of organic materials and polysaccharides including cellulose, sugars, chitin, lignin, starch and polylactic acids Zhang et al. (2000; Aira et al. 2007; Vivas et al. 2009). Single-strand conformation polymorphism (SSCP) profiles on the diversity of eight bacterial groups viz., Alphaproteobacteria, Betaproteobacteria, Bacteroidetes, Gammaproteobacteria, Deltaproteobacteria, Verrucomicrobia, Planctomycetes, and Firmicutes from fresh soil, gut, and casts of the earthworms *L. terrestris* and *Aporrectodea caliginosa* showed the presence of Bacteroidetes, Alphaproteobacteria, Betaproteobacteria and representatives of classes Flavobacteria, Sphingobacteria (Bacteroidetes) and *Pseudomonas* spp. in the worm casts in addition to unclassified Sphingomonadaceae (Alphaproteobacteria) and *Alcaligenes* spp. (Betaproteobacteria) (Nechitaylo et al. 2010).

### Role of vermicompost in soil fertility

Vermicomposts can significantly influence the growth and productivity of plants (Kale et al. 1992; Kalembasa 1996; Edwards 1988; Sinha et al. 2009) due to their micro and macro elements, vitamins, enzymes and hormones (Makulec 2002). Vermicomposts contain nutrients such as nitrates, exchangeable phosphorus, soluble potassium, calcium, and magnesium in plant available forms (Orozco et al. 1996; Edwards 1998) and have large particular surface area that provides many microsites for microbial activity and for the strong retention of nutrients (Shi-wei and Fu-zhen 1991). Uptake of nitrogen (N), phosphorus (P), potassium (K) and magnesium (Mg) by rice (*Oryza sativa*) plant was highest when fertilizer was applied in combination with vermicompost (Jadhav et al. 1997). N uptake by ridge gourd (*Luffa acutangula*) was higher when the fertilizer mix contained 50% vermicompost (Sreenivas et al. 2000). Apart from providing mineralogical nutrients, vermicomposts also contribute to the biological fertility by adding beneficial microbes to soil. Mucus, excreted through the earthworm`s digestive canal, stimulates antagonism and competition between diverse microbial populations resulting in the production of some antibiotics and hormone-like biochemicals, boosting plant growth (Edwards and Bohlen 1996). In addition, mucus accelerates and enhances decomposition of organic matter composing stabilized humic substances which embody water-soluble phytohormonal elements (Edwards and Arancon 2004) and plant-available nutrients at high levels (Atiyeh et al. 2000c). Adding vermicasts to soil improves soil structure, fertility, plant growth and suppresses diseases caused by soil-borne plant pathogens, increasing crop yield (Chaoui et al. 2002; Scheuerell et al. 2005; Singh et al. 2008). Kale (1995) reported the nutrient status of vermicomposts with organic carbon 9.15-17.98%, total nitrogen 0.5-1.5%, available phosphorus 0.1-0.3%, available potassium 0.15%, calcium and magnesium 22.70-70 mg/100 g, copper 2–9.3 ppm, zinc 5.7-11.5 ppm and available sulphur 128–548 ppm.

Effects of a variety of vermicomposts on a wide array of field crops (Chan and Griffiths 1988; Arancon et al. 2004b), vegetable plants (Edwards and Burrows 1988; Wilson and Carlile 1989;Subler et al. 1998; Atiyeh et al. 2000b), ornamental and flowering plants (Edwards and Burrows 1988; Atiyeh et al. 2000c) under greenhouse and field conditions have been documented. Vermicomposts are used as alternative potting media due to their low-cost, excellent nutrient status and physiochemical characters. Considerable improvements in plant growth recorded after amending soils with vermicomposts have been attributed to the physico-chemical and biological properties of vermicomposts.

Vermicompost addition favorably affects soil pH, microbial population and soil enzyme activities (Maheswarappa et al. 1999) and also reduces the proportion of water-soluble chemical, which cause possible environmental contamination (Mitchell and Edwards 1997). Vermicompost addition increases the macropore space ranging from 50–500 μm, resulting in improved air-water relationship in the soil, favourably affecting plant growth (Marinari et al. 2000). Evaluation of various organic and inorganic amendments on growth of raspberry proves that vermicompost has beneficial buffering capability and ameliorate the damage caused by excess of nutrients which may otherwise cause phytotoxicity (Subler et al. 1998). Thus, vermicompost acts a soil conditioner (Albanell et al. 1988) and a slow-release fertilizer (Atiyeh et al. 2000a). During vermicomposting the heavy metals forms complex, aggregates with humic acids and other polymerized organic fractions resulting in lower availability of heavy metals to the plant, which are otherwise phytotoxic (Dominguez and Edwards 2004). Soil amended with vermicompost produced better quality fruits and vegetables with less content of heavy metals or nitrate, than soil fertilized with mineral fertilizers (Kolodziej and Kostecka 1994).

### Role of vermicompost bacteria in biomedical waste management

The importance of sewage sludge, biosolids and biomedical waste management by safe, cheap and easy methods need no further emphasis. All these wastes are infectious and have to be disinfected before being disposed into the environment. Biosolids also contain an array of pathogenic microorganisms (Hassen et al. 2001). Biocomposting of wastes bring about biological transformation and stabilization of organic matter and effectively reduces potential risks of pathogens (Burge et al. 1987; Gliotti et al. 1997; Masciandaro et al. 2000). Vermicomposting does not involves a thermophilic phase which might increase the risk of using this technology for management of infectious wastes, but surprisingly vermicomposting resulted into a noticeable reduction in the pathogen indicators such as fecal coliform, *Salmonella* sp., enteric virus and helminth ova in the biosolids (Eastman 1999; Sidhu et al. 2001). Vermicomposting of biosolids resulted in reduction of faecal coliforms and *Salmonella* sp. from 39,000 MPN/g to 0 MPN/g and < 3 MPN to < 1MPN/g respectively (Dominguez and Edwards 2004). Vermicomposting of municipal sewage sludge with *L. mauritii* eliminated *Salmonella* and *Escherichia* sp., and the earthworm gut analysis also proved that *Salmonella* sp. ranging 15–17 × 10^3^ CFU/g and *Escherichia* sp. ranging 10–14 × 10^2^ CFU/g were completely eliminated in the gut after 70 days of vermicomposting period (Ganesh Kumar and Sekaran 2005). Activities by earthworms on sludge reduced levels of pathogens and odors of putrefaction and accelerated sludge stabilization (Mitchell 1978; Brown and Mitchell 1981; Hartenstein 1983). The reduction or removal of these enteric bacterial populations at the end of vermicomposting period, correlates with the findings that earthworm’s diet include microorganisms and earthworms ability to selectively digest them (Bohlen and Edwards 1995; Edwards and Bohlen 1996). Apart from solid waste management, earthworms are also used in sewage water treatment. Earthworms promote the growth of ‘beneficial decomposer bacteria’ in wastewater and acts as aerators, grinders, crushers, chemical degraders, and biological stimulators (Dash 1978; Sinha et al. 2002). Earthworms also granulate the clay particles and increase the hydraulic conductivity and natural aeration and further grind the silt and sand particles and increase the total specific surface area and thereby enhance adsorption of the organic and inorganic matter from the wastewater. In addition, earthworms body acts as a ‘biofilter’ and remove the biological oxygen demand (BOD), chemical oxygen demand (COD), total dissolved solids (TDS) and total suspended solids (TSS) from wastewater by 90%, 80–90%, 90–92% and 90–95% respectively by ‘ingestion’ and biodegradation of organic wastes, heavy metals, and solids from wastewater and by their ‘absorption’ through body walls (Sinha et al. 2008).

Reports reveal that vermicomposting converts the infected biomedical waste containing various pathogens viz., *Staphylococcus aureus*, *Proteus vulgaris*, *Pseudomonas pyocyaneae* and *Escherichia coli* to an innocuous waste containing commensals like *Citrobactor freundii* and aerobic spore bearing microorganism usually found in the soil and alimentary canal of earthworms (Umesh et al. 2006). Vermicomposting plays a vital role for safe management of biomedical wastes and solid wastes generated from wastewater treatment plants and its bioconversion into valuable composts free from enteric bacterial populations. Depending on the earthworm species, vermicomposting was known to reduce the level of different pathogens such as *Salmonella enteriditis, Escherichia coli,* total and faecal coliforms, helminth ova and human viruses in different types of waste. Direct means of reduction in these microbial numbers during gut passage might be due to the digestive enzymes and mechanical grinding, while indirect means of pathogen removal might be due to promotion of aerobic conditions which could bring down the load of coliforms (Monroy et al. 2009; Edwards 2011; Aira et al. 2011).

### Role of vermicompost in plant growth promotion

Use of vermicomposts as biofertilizers has been increasing recently due to its extraordinary nutrient status, and enhanced microbial and antagonistic activity. Vermicompost produced from different parent material such as food waste, cattle manure, pig manure, etc., when used as a media supplement, enhanced seedling growth and development, and increased productivity of a wide variety of crops (Edwards and Burrows 1988; Wilson and Carlile 1989; Buckerfield and Webster 1998; Edwards 1998; Subler et al. 1998; Atiyeh et al. 2000c). Vermicompost addition to soil-less bedding plant media enhanced germination, growth, flowering and fruiting of a wide range of green house vegetables and ornamentals (Atiyeh et al. 2000a, b, c), marigolds (Atiyeh et al. 2001), pepper (Arancon et al. 2003a), strawberries (Arancon et al. 2004b) and petunias (Chamani et al. 2008). Vermicompost application in the ratio of 20:1 resulted in a significant and consistent increase in plant growth in both field and greenhouse conditions (Edwards et al. 2004), thus providing a substantial evidence that biological growth promoting factors play a key role in seed germination and plant growth (Edwards and Burrows 1988; Edwards 1998). Investigations revealed that plant hormones and plant-growth regulating substances (PGRs) such as auxins, gibberellins, cytokinins, ethylene and abscisic acid are produced by microorganisms (Barea et al. 1976; Arshad and Frankenberger 1993).

Several researchers have documented the presence of plant growth regulators such as auxins, gibberellins, cytokinins of microbial origin (Krishnamoorthy and Vajranabhiah 1986; Grappelli et al. 1987; Tomati et al. 1988; Muscolo et al. 1999) and humic acids (Senesi et al. X^1992^; Masciandaro et al. 1997; Atiyeh et al. 2002) in vermicompost in appreciable quantities. Cytokinins produced by *Bacillus* and *Arthrobacter* spp. in soils increase the vigour of seedlings (Inbal and Feldman 1982; Jagnow 1987). Microbially produced gibberellins influence plant growth and development (Mahmoud et al. 1984; Arshad and Frankenberger 1993) and auxins produced by *Azospirillum brasilense* affects the growth of plants belonging to paoceae (Barbieri et al. 1988). Extensive investigations on the biological activities of humic substances showed that they also posses plant growth stimulating property (Chen and Aviad 1990). Humic substances increased the dry matter yields of corn and oat seedlings (Lee and Bartlett 1976; Albuzio et al. 1994); number and length of tobacco roots (Mylonas and Mccants 1980); dry weights of roots, shoots and number of nodules of groundnut, soyabean and clover plants (Tan and Tantiwiramanond 1983) and vegetative growth of chicory plants (Valdrighi et al. 1996) and induced root and shoot formation in plant tissue culture (Goenadi and Sudharama 1995). High levels of humus have been reported from vermicomposts originating from food wastes, animal manure, sewage and paper mill sludges (Atiyeh et al. 2002; Canellas et al. 2002; Arancon et al. 2003c). The humic and fulvic acid in the humus dissolves insoluble minerals in the organic matter and makes them readily available to plants and in addition they also help plants to overcome stress and stimulates plant growth (Sinha et al. 2010). Studies on biological activities of vermicompost derived humic substances, revealed that they had similar growth-promoting hormonal effect (Dell'Agnola and Nardi 1987; Nardi et al. 1988; Muscolo et al. 1993). The humic materials extracted from vermicomposts have been reported to produce auxin-like cell growth and nitrate metabolism in carrots (*Daucus carota*) (Muscolo et al. 1996). Humates obtained from pig manure vermicompost increased growth of tomato (Atiyeh et al. 2002) and those obtained from cattle, food and paper waste vermicompost increased the growth of strawberries and peppers (Arancon et al. 2003a).

Earthworms produce plant growth regulators (Gavrilov 1963). Since earthworms increase the microbial activity by several folds they are considered as important agents which enhance the production of plant growth regulators (Nielson 1965; Graff and Makeschin 1980; Dell'Agnola and Nardi 1987; Grappelli et al. 1987; Tomati et al. 1987, 1988; Edwards and Burrows 1988; Nardi et al. 1988; Edwards 1998). Plant growth stimulating substances of microbial origin were isolated from tissues of *Aporrectodea longa, L. terrestris* and *Dendrobaena rubidus* and indole like substances were detected from the tissue extracts of *A. caliginosa, L. rubellus* and *E. foetida* which increased the growth of peas (Nielson 1965) and dry matter production of rye grass (Graff and Makeschin 1980). *A. trapezoids* aided in the dispersal of *Rhizobium* through soil resulting in increased root colonization and nodulation of leguminous plants (Bernard et al. 1994). Use of earthworm casts in plant propagation promoted root initiation, increased root numbers and biomass. The hormone-like effect produced by earthworm casts on plant metabolism, growth and development causing dwarfing, stimulation of rooting, internode elongation and precociousness of flowering was attributed to the fact of presence of microbial metabolites (Tomati et al. 1987; Edwards 1998). Earthworm casts stimulated growth of ornamental plants and carpophore formation in *Agaricus bisporus* when used as casing layer in mushroom cultivation (Tomati et al. 1987). Aqueous extracts of vermicompost produced growth comparable to the use of hormones such as auxins, gibberellins and cytokinins on Petunia, Begonia and Coleus, providing solid evidence that vermicompost is a rich source of plant growth regulating substances (Grappelli et al. 1987; Tomati et al. 1987, 1988). Addition of vermicompost at very low levels to the growth media dramatically increased the growth of hardy ornamentals *Chamaecyparis lawsonian, Elaeagnus pungens, Pyracantha* spp., *Viburnum bodnantense, Cotoneaster conspicus* and *Cupressocyparis leylandi*. Cucumber (Hahn and Bopp 1968), dwarf maize (Sembdner et al. 1976) and coleus bioassays (Edwards et al. 2004) evidenced that vermicompost contained appreciable amounts of cytokinins, gibberellins and auxins respectively. Maize seedlings dipped in vermicompost water showed marked difference in plumule length compared to normal water indicating that plant growth promoting hormones are present in vermicompost (Nagavallemma et al. 2004). Comparative studies on the impact of vermiwash and urea solution on seed germination, root and shoot length in *Cyamopsis tertagonoloba* proved that vermiwash contained hormone like substances (Suthar 2010). High performance liquid chromatography (HPLC) and gas chromatography-mass spectroscopy (GC-MS) analyses of aqueous extracts of cattle waste derived vermicompost showed presence of significant amounts indole-acetic-acid (IAA), gibberellins and cytokinins (Edwards et al. 2004).

Earthworm gut associated microbes enrich vermicomposts with highly water-soluble and light-sensitive plant growth hormones, which gets absorbed on humic acid substances in vermicompost making them extremely stable and helps them persist longer in soils thereby influencing plant growth (Atiyeh et al. 2002; Arancon et al. 2003c). This is confirmed by presence of exchangeable auxin group in the macrostructure of humic acid extract from vermicompost (Canellas et al. 2002). Apart from the rich nutritional status and ready nutrient availability, presence of humic acids and plant growth regulating substances makes vermicompost a biofertilizer which increases germination, growth, flowering and fruiting in a wide range of crops. Vermicompost substitution in a relatively small proportion (10–20%) to the potting mixture increased dry matter production and tomato growth significantly (Subler et al. 1998). Soil amended with 20% vermicompost was more suitable for tomato seedling production (Valenzuela et al. 1997). Similarly vermicompost addition upto 50% in the medium resulted in enhanced growth of *Chamaecyparis lawsoniana* (Lawson's Cypress), *Juniperus communis* (Juniper) and *Elaeagnus pungens* (Silverberry) rooted liners (Bachman and Edgar Davice 2000).

Vermicompost application increased plant spread (10.7%), leaf area (23.1%), dry matter (20.7%) and increased total strawberry fruit yield (32.7%) (Singh et al. 2008). Substitution of vermicompost drastically reduced the incidence of physiological disorders like albinism (16.1–4.5%), fruit malformation (11.5–4.0%) and occurrence of grey mould (10.4–2.1%) in strawberry indicating its significance in reducing nutrient-related disorders and *Botrytis* rot, thereby increasing the marketable fruit yield upto 58.6% with better quality parameters. Fruit harvested from plant receiving vermicompost were firmer, had higher total soluble solids (TSS), ascorbic acid content and attractive colour. All these parameters appeared to be dose dependent and best results were achieved at 7.5 t ha^_1^ (Singh et al. 2008). Vermicompost application showed significant increase in germination percent (93%), growth and yield of mung bean (*Vigna radiata*) compared to the control (Karmegam et al. 1999). Similarly, the fresh and dry matter yields of cowpea (*Vigna unguiculata*) were higher in soil amended with vermicompost than with biodigested slurry, (Karmegam and Daniel 2000). Combined application of vermicompost with N fertilizer gave higher dry matter (16.2 g plant^-1^) and grain yield (3.6 t ha^-1^) of wheat (*Triticum aestivum*) and higher dry matter yield (0.66 g plant^-1^) of the following coriander (*Coriandrum sativum*) crop in wheat-coriander cropping system (Desai et al. 1999). Vermicompost application produced herbage yields of coriander cultivars comparable to those obtained with chemical fertilizers (Vadiraj et al. 1998). Yield of pea (*Pisum sativum*) increased with the application of vermicompost (10 t ha^-1^) along with recommended NPK (Meena et al. 2007). Vermicompost application to sorghum (*Sorghum bicolor*) (Patil and Sheelavantar 2000), sunflower (*Helianthus annuus*) (Devi et al. 1998), tomato (*Lycopersicon esculentum*) (Nagavallemma et al. 2004), eggplant (*Solanum melangona*) (Guerrero and Guerrero, 2006), okra (*Abelmoschus esculentus*) (Gupta et al. 2008), hyacinth bean (*Lablab purpureas*) (Karmegam and Daniel 2008), grapes (Buckerfield and Webster 1998) and cherry (Webster 2005) showed a positive result. Vermicompost amendment at the rate of 10 t ha^-1^ along with 50% of recommended dose of NPK fertilizer increased the number and fresh weight of flowers per plant, flower diameter and yield, while at the rate of 15 t ha^-1^ along with 50% of recommended dose of NPK increased vase life of *Chrysanthemum chinensis* (Nethra et al. 1999). Red Clover and cucumber grown in soil amended with vermicompost showed an increase in mineral contents viz., Ca, Mg, Cu, Mn and Zn in their shoot tissues (Sainz et al. 1998). Vermicomposted cow manure stimulated the growth of lettuce and tomato plants while the unprocessed parent material did not (Atiyeh et al. 2000b). Similarly, vermicomposted duck wastes resulted in better growth of tomatoes, lettuce, and peppers than the unprocessed wastes (Wilson and Carlile 1989). Theenhancement in plant growth might be attributed to the fact that processed waste had improved physicochemical characteristics and nutrients, in forms readily available to the plant as well as the presence of plant growth promoting and antagonistic disease suppressing beneficial bacteria.

### Role of vermicompost in plant disease management

#### Plant pathogen control

Soils with low organic matter and microbial activity are conducive to plant root diseases (Stone et al. 2004) and addition of organic amendments can effectively suppress plant disease (Raguchander et al. 1998; Blok et al. 2000; Lazarovits et al. 2000). Several researchers reported the disease suppressive properties of thermophilic compost (Hoitink et al. 1997; Goldstein 1998; Pitt et al. 1998) on a wide range of phytopathogens viz., *Rhizoctonia* (Kuter et al. 1983), *Phytopthora* (Hoitink and Kuter 1986; Pitt et al. 1998), *Plasmidiophora brassicae* and *Gaeumannomyces graminis* (Pitt et al. 1998) and *Fusarium* (Kannangowa et al. 2000; Cotxarrera et al. 2002). Microbial antagonism might be one of the possible reasons for disease suppression as organic amendments enhances the microbial population and diversity. Traditional thermophilic composts promote only selected microbes while non-thermophilic vermicomposts are rich sources of microbial diversity and activity and harbour a wide variety of antagonistic bacteria thus acts as effective biocontrol agents aiding in suppression of diseases caused by soil-borne phytopathogenic fungi (Chaoui et al. 2002; Scheuerell et al. 2005; Singh et al. 2008).

Earthworm feeding reduces the survival of plant pathogens such as *Fusarium* sp. and *Verticillium dahliae* (Yeates 1981; Moody et al. 1996) and increases the densities of antagonistic fluorescent pseudomonads and filamentous actinomycetes while population densities of *Bacilli* and *Trichoderma* spp. remains unaltered (Elmer 2009). Earthworm activities reduce root diseases of cereals caused by *Rhizoctonia* (Doube et al. 1994). It has been proved that earthworms decreased the incidence of field diseases of clover, grains, and grapes incited by *Rhizoctonia* spp. (Stephens et al. 1994a; Stephens and Davoren 1997) and *Gaeumannomyces* spp. (Clapperton et al. 2001). Earthworms *Aporrectodea trapezoides* and *Aporrectodea rosea* act as vectors of *Pseudomonas corrugata 2*14OR, a biocontrol agent for wheat take-all caused by *G. graminis* var. *tritd* (Doube et al. 1994). Greenhouse studies on augmentation of pathogen infested soils with *L. terrestris* showed a significant reduction of disease caused by *Fusarium oxysporum* f. sp. *asparagi* and *F. proliferatum* on susceptible cultivars of asparagus (*Asparagus officinalis*), *Verticillium dahliae* on eggplant (*Solanum melongena*) and *F. oxysporum* f. sp*. lycopersici* race 1 on tomato. Plant weights increased by 60-80% and disease severity reduced by 50-70% when soils were augmented with earthworms. Incorporation of soil with vermicompost effectively suppressed *R. solani* in wheat (Stephens et al. 1993), *Phytophthora nicotianae* (Nakamura 1996; Szczech 1999; Szczech and Smolinska 2001) and *Fusarium* in tomatoes (Nakamura 1996; Szczech 1999), *Plasmodiophora brassicae* in tomatoes and cabbage (Nakamura 1996), *Pythium* and *Rhizoctonia* (root rot) in cucumber and radish (Simsek Ersahin et al. 2009), *Botrytis cineria* (Singh et al. 2008) and *Verticillium* (Chaoui et al. 2002) in strawberry and *Sphaerotheca fulginae* in grapes (Edwards et al. 2004). Vermicompost application drastically reduced the incidence of ‘Powdery Mildew’, ‘Color Rot’ and ‘Yellow Vein Mosaic’ in Lady’s finger (*Abelmoschus esculentus*) (Agarwal et al. 2010). Substitution of vermicompost in the growth media reduced the fungal diseases caused by *R. solani, P. drechsleri and F. oxysporum* in gerbera (Rodriguez et al. 2000). Amendment of vermicompost at low rates (10-30%) in horticulture bedding media resulted in significant suppression of *Pythium* and *Rhizoctonia* under green house conditions (Edwards et al. 2004). Research findings proved that vermicompost when added to container media significantly reduced the infection of tomato plants by *P. nicotianae* var. nicotianae and *F. oxysporum* sp. *lycopersici* (Szcech et al. 1993; Szczech 1999). Club-rot of cabbage caused by *P. brassicae* was inhibited by dipping cabbage roots into a mixture of clay and vermicompost (Szcech et al. 1993). Potato plants treated with vermicompost were less susceptible to *P. infestans* than plants treated with inorganic fertilizers (Kostecka et al. 1996a). Aqueous extracts of vermicompost inhibited mycelial growth of *B. cineria*, *Sclerotinia sclerotiorum, Corticium rolfsii*, *R. solani* and *F. oxysporum* (Nakasone et al. 1999), effectively controlled powdery mildew of barley (Weltzien 1989) and affected the development of powdery mildews on balsam (*Impatiens balsamina*) and pea (*Pisum sativum*) caused by *Erysiphe cichoracearum* and *E. pisi,* respectively in field conditions (Singh et al. 2003).

#### Mechanisms that mediate pathogen suppression

Two possible mechanisms of pathogen suppression have been described, one depends on systemic plant resistance and the other is mediated by microbial competition, antibiosis and hyperparasitism (Hoitink and Grebus 1997). The microbially mediated suppression is again classified into two mechanisms viz., ‘general suppression’ where a wide range of microbes suppress the pathogens such as *Pythium* and *Phytopthora* (Chen et al. 1987) and ‘specific suppression’ where a narrow range of organisms facilitates suppression, for instance disease caused by *Rhizoctonia* (Hoitink et al. 1997). The disease suppressive effect of vermicompost against fusarium wilt of tomato clearly depicted that fungus inhibition was purely biotic and no chemical factors played any role, since the experiments with heat-sterilized vermicompost failed to control the disease (Szczech 1999). Experiments on suppression of damping-off caused by *R. solani,* in vermicompost amended nurseries of white pumpkin proved that vermicompost suppressed the disease in a dosage and temperature dependent manner (Rivera et al. 2004). Earthworm castings are rich in nutrients (Lunt and Jacobson 1944; Parle 1963) and calcium humate, a binding agent (Edwards 1998) that reduces desiccation of individual castings and favors the incubation and proliferation of beneficial microbes, such as *Trichoderma* spp. (Tiunov and Scheu 2000), *Pseudomonas* spp. (Schmidt et al. 1997), and mycorrhizal spores (Gange 1993; Doube et al. 1995). Earthworm activity increased the communities of Gram-negative bacteria (Clapperton et al. 2001; Elmer 2009). Vermicompost associated chitinolytic bacterial communities viz., *Nocardioides oleivorans*, several species of *Streptomyces* and *Staphylococcus epidermidis* showed inhibitory effects against plant phytopathogens such as, *R. solani, Colletotrichum coccodes, Pythium ultimum, P. capsici* and *Fusarium moniliforme* (Yasir et al. 2009a).

#### Role of vermicompost in arthropod pest control

Addition of organic amendments helped in suppression of various insect pests such as European corn borer (Phelan et al. 1996), other corn insect pests (Biradar et al. 1998), aphids and scale insects (Culliney and Pimentel 1986; Costello and Altiei 1995; Huelsman et al. 2000) and brinjal shoot and fruit borer (Sudhakar et al. 1998). Several reports also evidenced that vermicompost addition decreased the incidence of *Spodoptera litura, Helicoverpa armigera*, leaf miner (*Apoaerema modicella)*, jassids (*Empoasca kerri*), aphids (*Aphis craccivora)* and spider mites on groundnuts (Rao et al. 2001; Rao 2002, 2003) and psyllids (*Heteropsylla cubana*) on a tropical leguminous tree (*Leucaena leucocephala*) (Biradar et al. 1998). Vermicompost amendment decreased the incidence of sucking pests under field conditions (Ramesh 2000) and suppressed the damage caused by of two-spotted spider mite (*Tetranychus* spp.), aphid (*Myzus persicae*) (Edwards et al. 2007) and mealy bug (*Pseudococcus* spp.) under green house conditions (Arancon et al. 2007). Vermicompost substitution to soil less plant growth medium MetroMix 360 (MM360) at a rate less then 50% reduced the damage caused by infestation of pepper seedlings by *M. persicae* and *Pseudococcus* spp. and tomato seedlings by *Pseudococcus* spp., cabbage seedlings by *M. persicae* and cabbage white caterpillars (*Pieris brassicae* L.) (Arancon et al. 2005). Greenhouse cage experiments conducted on tomatoes and cucumber seedlings infested with *M. persicae*, citrus mealybug (*Planococcus citri*), two spotted spider mite (*Tetranychus urticae*); striped cucumber beetles (*Acalymna vittatum*) attacking cucumbers and tobacco hornworms (*Manduca sexta*) attacking tomatoes proved that treatment of infested plants with aqueous extracts of vermicompost suppressed pest establishment, and their rates of reproduction. Vermicompost teas at higher dose also brought about pest mortality (Edwards et al. 2010b). Suppression of aphid population gains importance since they are key vectors in transmission of plant viruses. Addition of solid vermicompost reduced damage by *A. vittatum* and spotted cucumber beetles (*Diabotrica undecimpunctata*) on cucumbers and larval hornworms (*Manduca quinquemaculata)* on tomatoes in both greenhouse and field experiments (Yardim et al. 2006). Combined application of vermicompost and vermiwash spray to chilli (*Capiscum annum*) significantly reduced the incidence of ‘Thrips’ (*Scirtothrips dorsalis*) and ‘Mites’ (*Polyphagotarsonemus latus*) (Saumaya et al. 2007).

#### Mechanisms that mediate pest control

Plants grown in inorganic fertilizers are more prone to pest attack than those grown on organic fertilizers (Culliney and Pimentel 1986; Yardim and Edwards 2003; Phelan 2004). Inorganic nitrogen fertilization improves the nutritional quality and palatability of the host plants, inhibits the raise of secondary metabolite concentrations (Fragoyiannis et al. 2001; Herms 2002), enhances the fecundity of insects dieting on them, attracts more individuals for oviposition (Bentz et al. 1995) and increases the population growth rates of insects (Culliney and Pimentel 1986; Jannsson and Smilowitz 1986). Though organic fertilizer has an enhanced nutritional composition they release nutrients at a slower rate (Patriquin et al. 1995) hence plants grown with organic fertilizers possess decreased N levels (Steffen et al. 1995) and have higher phenol content (Asami et al. 2003) resulting in resistance of these plants to pest attack. Similarly vermicomposts exhibit a slow, balanced nutritional release pattern, particularly in release of plant available N, soluble K, exchangeable Ca, Mg and P (Edwards and Fletcher 1988; Edwards 1998). Vermicomposts are rich in humic acid and phenolic compounds. Phenolic compounds act as feeding deterrents and hence significantly affect pest attacks (Kurowska et al. 1990; Summers and Felton 1994; QiTian 2004; Hawida et al. 2007; Koul 2008; Mahanil et al. 2008; Bhonwong et al. 2009). Soil containing earthworms contained polychlorinated phenols and their metabolites (Knuutinen et al. 1990). An endogenous phenoloxidase present in *L. rubellus* bioactivate compounds to form toxic phenols viz., *p*-nitrophenol (Park et al. 1996). Monomeric phenols could be absorbed by humic acids in the gut of earthworms (Vinken et al. 2005). Uptake of soluble phenolic compounds from vermicompost, by the plant tissues makes them unpalatable thereby affecting pest rates of reproduction and survival (Edwards et al. 2010a; Edwards et al. 2010b).

#### Role of vermicompost in nematode control

It has been well documented that addition of organic amendments decreases the populations of plant parasitic nematodes (Addabdo 1995; Sipes et al. 1999; Akhtar and Malik 2000). Vermicompost amendments appreciably suppress plant parasitic nematodes under field conditions (Arancon et al. 2003b). Vermicomposts also suppressed the attack of *Meloidogyne incognita* on tobacco, pepper, strawberry and tomato (Swathi et al. 1998; Edwards et al. 2007; Arancon et al. 2002; Morra et al. 1998) and decreased the numbers of galls and egg masses of *Meloidogyne javanica* (Ribeiro et al. 1998).

#### Mechanisms that mediate nematode control

There are several feasible mechanisms that attribute to the suppression of plant parasitic nematodes by vermicompost application and it involves both biotic and abiotic factors. Organic matter addition to the soil stimulates the population of bacterial and fungal antagonists of nematodes (*e.g*., *Pasteuria penetrans*, *Pseudomonas* spp. and chitinolytic bacteria, *Trichoderma* spp.), and other typical nematode predators including nematophagous mites viz., *Hypoaspis calcuttaensis* (Bilgrami 1996), Collembola and other arthropods which selectively feeds on plant parasitic nematodes. (Thoden et al. 2011). Vermicompost amendment promoted fungi capable of trapping nematode and destroying nematode cysts (Kerry 1988) and increased the population of plant growth-promoting rhizobacteria which produce enzymes toxic to plant parasitic nematodes (Siddiqui and Mahmood 1999). Vermicompost addition to soils planted with tomatoes, peppers, strawberry and grapes showed a significant reduction of plant parasitic nematodes and increased the population of fungivorous and bacterivorous nematodes compared to inorganic fertilizer treated plots (Arancon et al. 2002). In addition, few abiotic factors viz., nematicidal compounds such as hydrogen sulphide, ammonia, nitrates, and organic acids released during vermicomposting, as well as low C/N ratios of the compost cause direct adverse effects while changes in soil physiochemical characterists viz., bulk density, porosity, water holding capacity, pH, EC, CEC and nutrition posses indirect adverse effects on plant parasitic nematodes (Rodriguez-Kabana 1986; Thoden et al. 2011).

## Conclusion

Vermicomposting is a cost-effective and eco-friendly waste management technology which takes the previlige of both earthworms and the associated microbes and has many advantages over traditional thermophilic composting. Vermicomposts are excellent sources of biofertilizers and their addition improves the physiochemical and biological properties of agricultural soil. Vermicomposting amplifies the diversity and population of beneficial microbial communities. Although there are some reports indicating that few harmful microbes such as spores of *Pythium* and *Fusarium* are dispersed by earthworms (Edwards and Fletcher 1988), the presence and amplification of antagonistic disease-suppressing and other plant growth-promoting beneficial bacteria during vermicomposting out weigh these harmful effects (Edwards and Fletcher 1988; Gammack et al. 1992; Brown 1995). Vermicomposts with excellent physio-chemical properties and buffering ability, fortified with all nutrients in plant available forms, antagonistic and plant growth-promoting bacteria are fantabulous organic amendments that act as a panacea for soil reclamation, enhancement of soil fertility, plant growth, and control of pathogens, pests and nematodes for sustainable agriculture.

## References

[CR1_31] Addabdo TD (1995). The nematicidal effect of organic amendments: a review of the literature 1982–1994. Nematol Mediterranea.

[CR2_31] Aira M, Monroy F, Dominguez J (2007). Earthworms strongly modify microbial biomass and activity triggering enzymatic activities during vermicomposting independently of the application rates of pig slurry. Sci Total Environ.

[CR3_31] Aira M, Gómez-Brandón M, González-Porto P, Domínguez J (2011). Selective reduction of the pathogenic load of cow manure in an industrial-scale continuous-feeding vermireactor. Bioresource Technol.

[CR4_31] Agarwal S, Sinha RK, Sharma J, Sinha RK (2010). Ver-miculture for sustainable horticulture: Agronomic impact studies of earthworms, cow dung compost and vermi-compost vis-à-vis chemical fertilizers on growth and yield of lady’s finger (Abelmoschus esculentus). Special Issue on ‘Vermiculture Technology’, International Journal of Environmental Engineering.

[CR5_31] Akhtar M, Malik A (2000). Role of organic amendments and soil organisms in the biological control of plant parasitic nematodes: a review. Bioresour Technol.

[CR6_31] Albanell E, Plaixats J, Cabrero T (1988). Chemical changes during vermicomposting (Eisenia fetida) of sheep manure mixed with cotton industrial wastes. Biol Fertil Soils.

[CR7_31] Albuzio A, Concheri G, Nardi S, Dell’Agnola G, Senesi N, Miano TM (1994). Effect of humic fractions of different molecular size on the development of oat seedlings grown in varied nutritional conditions. Humic substances in the Global Environment and Implications on Human Health.

[CR8_31] Arancon NQ, Edwards CA, Atiyeh R, Metzger JD (2004). Effects of vermicomposts produced from food waste on the growth and yields of greenhouse peppers. Bioresour Technol.

[CR9_31] Arancon NQ, Edwards CA, Bierman P, Metzger JD, Lee S, Welch C (2003). Effects of vermicomposts to tomatoes and peppers grown in the field and strawberries under high plastic tunnels. Pedobiologia.

[CR10_31] Arancon NQ, Edwards CA, Bierman P, Welch C, Metzger JD (2004). The influence of vermicompost applications to strawberries: Part 1. Effects on growth and yield. Bioresour Technol.

[CR11_31] Arancon NQ, Edwards CA, Lee S (2002). Management of plant parasitic nematode populations by use of vermicomposts. Proceedings Brighton Crop Protection Conference – Pests and Diseases.

[CR12_31] Arancon NQ, Edwards CA, Yardim EN, Oliver TJ, Byrne RJ, Keeney G (2007). Suppression of two-spotted spider mite (Tetranychus urticae), mealy bug (Pseudococcus sp) and aphid (Myzus persicae) populations and damage by vermicomposts. Crop Prot.

[CR13_31] Arancon NQ, Galvis PA, Edwards CA (2005). Suppression of insect pest populations and damage to plants by vermicomposts. Bioresour Technol.

[CR14_31] Arancon NQ, Galvis P, Edwards CA, Yardim E (2003). The trophic diversity of nematode communities in soils treated with vermicomposts. Pedobiologia.

[CR15_31] Arancon NQ, Lee S, Edwards CA, Atiyeh RM (2003). Effects of humic acids and aqueous extracts derived from cattle, food and paper-waste vermicomposts on growth of greenhouse plants. Pedobiologia.

[CR16_31] Arshad M, Frankenberger WT, Metting FB (1993). Microbial production of plant growth regulators. Soil Microbial Ecology: Applications in Agricultural and Environmental Management.

[CR17_31] Asami DK, Hang YJ, Barnett DM, Mitchell AE (2003). Comparison of the total phenolic and ascorbic acid content of freeze-dried and air-dried marionberry, strawberry and corn grown using conventional organic and sustainable agricultural practices. J Agric Food Chem.

[CR18_31] Asha A, Tripathi AK, Soni P (2008). Vermicomposting: A Better Option for Organic Solid Waste Management. J Hum Ecol.

[CR19_31] Atiyeh RM, Arancon NQ, Edwards CA, Metzger JD (2001). The influence of earthworm-processed pig manure on the growth and productivity of marigolds. Bioresour Technol.

[CR20_31] Atiyeh RM, Dominguez J, Subler S, Edwards CA (2000). Changes in biochemical properties of cow manure during processing by earthworms (Eisenia andrei, Bouché) and the effects on seedling growth. Pedobiologia.

[CR21_31] Atiyeh RM, Lee S, Edwards CA, Arancon NQ, Metzger JD (2002). The influence of humic acids derived from earthworm-processed organic wastes on plant growth. Bioresour Technol.

[CR22_31] Atiyeh RM, Subler S, Edwards CA, Bachman G, Metzger JD, Shuster W (2000). Effects of vermicomposts and composts on plant growth in horticulture container media and soil. Pedobiologia.

[CR23_31] Atiyeh RM, Arancon NQ, Edwards CA, Metzger JD (2000). Influence of earthworm- processed pig manure on the growth and yield of green house tomatoes. Bioresour Technol.

[CR24_31] Ayyadurai N, Ravindra Naik P, Sakthivel N (2007). Functional characterization of antagonistic fluorescent pseudomonads associated with rhizospheric soil of rice (Oryza sativa L.). J Microbiol Biotechnol.

[CR25_31] Bachman GR, Edgar Davice W (2000). Growth of magnolia virginiana liners in vermicompost-amended media.

[CR26_31] Bansal S, Kapoor KK (2000). Vermicomposting of crop residues and cattle dung with Eisenia foetida. Bioresour Technol.

[CR27_31] Banu JR, Logakanthi S, Vijayalakshmi GS (2001). Biomanagement of paper mill sludge using an indigenous (Lampito mauritii) and two exotic (Eudrilus eugineae and Eisenia foetida) earthworms. J Environ Biol.

[CR28_31] Barbieri P, Bernardi A, Galli E, Zanetti G, Klingmüller W (1988). Effects of inoculation with different strains of Azospirillum brasilense on wheat roots development. Azospirillum IV, Genetics, Physiology, Ecology.

[CR29_31] Barea JM, Navarro E, Montana E (1976). Production of plant growth regulators by rhizosphere phosphate solubilizing bacteria. J Appl Bacteriol.

[CR30_31] Benitez E, Nogales R, Elvira C, Masciandaro G, Ceccanti B (1999). Enzymes activities as indicators of the stabilization of sewage sludges composting by Eisenia foetida. Bioresour Technol.

[CR31_31] Bentz JA, Reeves J, Barbosa P, Francis B (1995). Nitrogen fertilizer effect on selection, acceptance and suitability of Euphorbia pulcherrima (Euphorbiaceae) as a host plant to Bemisia tabaci (Homoptera: Aleyrodidae). Environ Entomol.

[CR32_31] Bernal MP, Faredes C, Sanchez-Monedero MA, Cegarra J (1998). Maturity and stability parameters of composts prepared with a, wide range of organic wastes. Bioresour Technol.

[CR33_31] Bernard MD, Peter MS, Christopher WD, Maarten HR (1994). Interactions between earthworms, beneficial soil microorganisms and root pathogens. Appl Soil Ecol.

[CR34_31] Bhatnagar RK, Palta RK (1996). Earthworm-Vermiculture and Vermicomposting.

[CR35_31] Bhonwong A, Stout MJ, Attajarusit J, Tantasawat P (2009). Defensive role of tomato polyphenoloxidases against cotton bollworm (Helicoverpa armigera) and beet army worm (Spodoptera exigua). J Chem Ecol.

[CR36_31] Bilgrami AL (1996). Evaluation of the predation abilities of the mite Hypoaspis calcuttaensis, predaceous on plant and soil nematodes. Fund Appl Nematol.

[CR37_31] Binet F, Fayolle L, Pussard M (1998). Significance of earthworms in stimulating soil microbial activity. Biol Fertil Soils.

[CR38_31] Biradar AP, Sunita ND, Teggel RG, Devaradavadgi SB (1998). Effect of vermicompost on the incidence of subabul psyllid. Insect- Environ.

[CR39_31] Blok WJ, Lamers JG, Termoshuizen AJ, Bollen GJ (2000). Control of soil-borne plant pathogens by incorporating fresh organic amendments followed by tarping. Phytopathology.

[CR40_31] Bohlen PJ, Edwards CA (1995). Earthworm effects on N dynamics and soil respiration in microcosms receiving organic and inorganic nutrients. Soil Biol Biochem.

[CR41_31] Brown BA, Mitchell MJ (1981). Role of the earthworm, Eisenia fetida, in affecting survival of Salmonella enteritidis ser. Typhimurium. Pedobiologia.

[CR42_31] Brown GG (1995). How do earthworms affect microfloral and faunal community diversity?. Plant Soil.

[CR43_31] Brown GG, Barois I, Lavelle P (2000). Regulation of soil organic matter dynamics and microbial activity in the drilosphere and the role of interactions with other edaphic functional domains. Eur J Soil Biol.

[CR44_31] Buckalew DW, Riley RK, Yoder WA, Vail WJ (1982). Invertebrates as vectors of endomycorrhizal fungi and Rhizobium upon surface mine soils. West Virginia Acad Sci Proc.

[CR45_31] Buckerfield JC, Webster KA (1998). Worm-worked waste boosts grape yields: prospects for vermicompost use in vineyards. Australian and New Zealand Wine Industry Journal.

[CR46_31] Burge WD, Enkiri NK, Hussong D (1987). Salmonella regrowth in compost as influenced by substrate. Microbial Ecol.

[CR47_31] Butt KR (1993). Utilization of solid paper mill sludge and spent brewery yeast as a feed for soil dwelling earthworms. Bioresour Technol.

[CR48_31] Byzov BA, Nechitaylo TY, Bumazhkin BK, Kurakov AV, Golyshin PN, Zvyagintsev DG (2009). Culturable microorganisms from the earthworm digestive tract. Microbiology.

[CR49_31] Canellas LP, Olivares FL, Okorokova FAR (2002). Humic acids isolated from earthworm compost enhance root elongation, lateral root emergence and plasma membrane H+− ATPase activity in maize roots. Plant Physiol.

[CR50_31] Chamani E, Joyce DC, Reihanytabar A (2008). Vermicompost Effects on the Growth and Flowering of Petunia hybrida ‘Dream Neon Rose’. American-Eurasian J Agric And Environ Sci.

[CR51_31] Chan LPS, Griffiths DA (1988). The vermicomposting of pretreated pig manure. Biol Wastes.

[CR52_31] Chaoui H, Edwards CA, Brickner M, Lee S, Arancon N (2002). Suppression of the plant diseases, Pythium (damping off), Rhizoctonia (root rot) and Verticillum (wilt) by vermicomposts. Proceedings of Brighton Crop Protection Conference – Pests and Diseases.

[CR53_31] Chen W, Hoitink HA, Schmitthenner AF, Touvinen O (1987). The role of microbial activity in suppression of damping off caused by Pythium ultimum. Phytopathology.

[CR54_31] Chen Y, Aviad T, MacCarthy P, Clapp CE, Malcolm RL, Bloom PR (1990). Effects of humic substances on plant growth. Humic Substances in Soil and Crop Sciences.

[CR55_31] Citernesi U, Neglia R, Seritti A, Lepidi AA, Filippi C, Bagnoli G, Nuti MP, Galluzzi R (1977). Nitrogen fixation in the gastro-enteric cavity of soil animals. Soil Biol Biochem.

[CR56_31] Clapperton MJ, Lee NO, Binet F, Conner RL (2001). Earthworms indirectly reduce the effect of take-all (Gaeumannomyces graminis var. tritici) on soft white spring wheat (Triticium aestivum cv. Fielder). Soil Biol Biochem.

[CR57_31] Contreras E (1980). Studies on the intestinal actinomycete flora of Eisenia lucens (Annelida, Oligochaeta). Pedobiologia.

[CR58_31] Correa JD, Barrios ML, Galdona RP (2004). Screening for plant growth promoting rhizobacteria in Chamaecytisus proliferus (tagasaste), a forage tree-shrub legume endemic to the Canary Islands. Plant Soil.

[CR59_31] Costello MJ, Altiei MA (1995). Abundance, growth rate and parasitism of Brevicoryne brassicae and Myzus persicae (Homoptera: Aphididae) on broccoli grown in living mulches. Agric Ecosyst Environ.

[CR60_31] Cotxarrera L, Trillas-Gayl MI, Steinberg C, Alabouvette C (2002). Use of sewage sludge compost and Trichoderma asperellum isolates to suppress Fusarium wilt of tomato. Soil Biol Biochem.

[CR61_31] Culliney TW, Pimentel D (1986). Ecological effects of organic agricultural practices on insect populations. Agric Ecosyst Environ.

[CR62_31] Daniel O, Anderson JM (1992). Microbial biomass and activity in contrasting soil material after passage through the gut of earthworm Lumbricus rubellus Hoffmeister. Soil Biol Biochem.

[CR63_31] Darwin F, Seward AC, John M (1903). More letters of Charles Darwin. A record of his work in series of hitherto unpublished letters.

[CR64_31] Dash MC, Singh JS, Gopal B (1978). Role of earthworms in the decomposer system. Glimpses of ecology.

[CR65_31] Dell'Agnola G, Nardi S (1987). Hormone-like effect and enhanced nitrate uptake induced by depolyconder humic fractions obtained from Allolobophora rosea and A. caliginosa faeces. Biol Fertil Soils.

[CR66_31] Desai VR, Sabale RN, Raundal PV (1999). Integrated nitrogen management in wheat-coriander cropping system. J Maharasthra Agric Univ.

[CR67_31] Devi D, Agarwal SK, Dayal D (1998). Response of sunflower (Helianthus annuus) to organic manures and fertilizers. Indian J Agron.

[CR68_31] Devliegher W, Verstraete W (1995). Lumbricus terrestris in a soil core experiment: nutrient-enrichment processes (NEP) and gut-associated processes (GAP) and their effect on microbial biomass and microbial activity. Soil Biol Biochem.

[CR69_31] Devliegher W, Verstraete W (1997). Microorganisms and soil physicochemical conditions in the drilosphere of Lumbricus terrestris. Soil Biol Biochem.

[CR70_31] Dominguez J, Edwards CA, Webster M (2000). Vermicomposting of sewage sludge: effects of bulking materials on the growth and reproduction of the earthworm Eisenia andrei. Pedobiologia.

[CR71_31] Dominguez J, Edwards CA, Shakir Hanna SH, Mikhail WZA (2004). Vermicomposting organic wastes: A review. Soil Zoology for sustainable Development in the 21st century.

[CR72_31] Doube BM, Ryder MH, Davoren CW, Meyer T (1995). Earthworms: a down under delivery system service for biocontrol agents of root disease. Acta Zool Fennica.

[CR73_31] Doube BM, Stephens PM, Davorena CW, Ryderb MH (1994). Interactions between earthworms, beneficial soil microorganisms and root pathogens. Appl Soil Ecol.

[CR74_31] Eastman BR (1999). Achieving pathogen stabilization using vermicomposting. BioCycle.

[CR75_31] Edwards CA, Edwards CA, Neuhauser EF (1988). Breakdown of animal, vegetable and industrial organic wastes by earthworms. Earthworms in Waste and Environmental Management SPB.

[CR76_31] Edwards CA, Edwards CA (1998). The use of earthworms in the breakdown and management of organic wastes. Earthworm Ecology.

[CR77_31] Edwards CA, Edwards CA, Arancon NQ, Sherman R (2011). Human pathogen reduction during vermicomposting. Vermiculture technology: earthworms, organic wastes and environmental management.

[CR78_31] Edwards CA, Arancon NQ, Bennett MV, Askar A, Keeney G, Little B (2010). Suppression of green peach aphid (Myzus persicae) (Sulz.), citrus mealybug (Planococcus citri) (Risso), and two spotted spider mite (Tetranychus urticae) (Koch.) attacks on tomatoes and cucumbers by aqueous extracts from vermicomposts. Crop Prot.

[CR79_31] Edwards CA, Arancon NQ, Bennett MV, Askar A, Keeney G (2010). Effect of aqueous extracts from vermicomposts on attacks by cucumber beetles (Acalymna vittatum) (Fabr.) on cucumbers and tobacco hornworm (Manduca sexta) (L.) on tomatoes. Pedobiologia.

[CR80_31] Edwards CA, Arancon NQ, Emerson E, Pulliam R (2007). Supressing plant parasitic nematodes and arthropod pests with vermicompost teas. Biocycle.

[CR81_31] Edwards CA, Arancon NQ (2004). Vermicomposts suppress plant pest and disease attacks. BioCycle.

[CR82_31] Edwards CA, Bohlen PJ (1996). Biology and Ecology of earthworms.

[CR83_31] Edwards CA, Burrows I, Fletcher KE, Jones BA, Gasser JKR (1985). The use of earthworms for composting farm wastes. Composting Agricultural and Other Wastes.

[CR84_31] Edwards CA, Burrows I, Edwards CA, Neuhauser E (1988). The potential of earthworm composts as plant growth media. Earthworms in Waste and Environmental Management.

[CR85_31] Edwards CA, Dominguez J, Arancon NQ, Shakir Hanna SH, Mikhail WZA (2004). The influence of vermicomposts on pest and diseases. Soil Zoology for Sustainable Development in the 21st centuary.

[CR86_31] Edwards CA, Dominguez J, Neuhauser EF (1998). Growth and reproduction of Perionyx excavatus (Perr.) (Megascolecidae) as factors in organic waste management. Biol Fertil Soils.

[CR87_31] Edwards CA, Fletcher KE (1988). Interaction between earthworms and microorganisms in organic matter breakdown. Agric Ecosyst Environ.

[CR88_31] Edwards CA, Lofty R (1977). The Biology of Earthworms.

[CR89_31] Elmer WH (2009). Influence of earthworm activity on soil microbes and soilborne diseases of vegetables. Plant Dis.

[CR90_31] Elvira C, Dominguez J, Sampedro L, Mato S (1995). Vermicomposting for the pulp industry. Biocycle.

[CR91_31] Elvira C, Sampedro L, Benítez E, Nogales R (1998). Vermicomposting of sludges from paper mill and dairy industries with Eisenia andrei: a pilot-scale study. Bioresour Technol.

[CR92_31] Elvira C, Sampedro L, Dominguez J, Mato S (1997). Vermicomposting of wastewater sludge from paper-pulp industry with nitrogen rich materials. Soil Biol Biochem.

[CR93_31] Fracchia L, Dohrmann AB, Martinotti MG, Tebbe CC (2006). Bacterial diversity in a finished compost and vermicompost: differences revealed by cultivation independent analyses of PCR-amplified 16S rRNA genes. Appl Microbiol Biotechnol.

[CR94_31] Fragoyiannis DA, McKinlay RG, D’Mello JPF (2001). Interactions of aphids herbivory and nitrogen availability on the total foliar glycoalkoloid content of potato plants. J Chem Ecol.

[CR95_31] Gajalakshmi S, Ramasamy EV, Abbasi SA (2001). Assessment of sustainable vermiconversion of water hyacinth at different reactor efficiencies employing Eudrilus engeniae Kingburg. Bioresour Technol.

[CR96_31] Gajalakshmi S, Ramasamy EV, Abbasi SA (2002). Vermicomposting of paper waste with the anecic earthworm Lampito mauritii Kingburg. Indian J Chem Technol.

[CR97_31] Gammack SM, Paterson E, Kemp JS, Cresser MS, Killham K, Stotzky G, Bolla LM (1992). Factors affecting movement of microorganisms in soils. Soil Biochemistry, 7.

[CR98_31] Gandhi M, Sangwan V, Kapoor KK, Dilbaghi N (1997). Composting of household wastes with and without earthworms. Environ Ecol.

[CR99_31] Ganesh kumar A, Sekaran G (2005). Enteric pathogen modification by anaecic earthworm, Lampito Mauritii. J Appl Sci Environ Mgt.

[CR100_31] Gange AC (1993). Translocation of mycorrhizal fungi by earthworms during early succession. Soil Biol Biochem.

[CR101_31] Garcia C, Hernandez T, Costa F (1997). Potential use of dehydrogenase activity as an index of microbial activity in degraded soils. Commun Soil Sci Plant Anal.

[CR102_31] Garg P, Gupta A, Satya S (2006). Vermicomposting of different types of waste using Eisenia foetida: a comparative study. Bioresour Technol.

[CR103_31] Gavrilov K (1963). Earthworms, producers of biologically active substances. Zh Obshch Biol.

[CR104_31] Ghosh M, Chattopadhyay GN, Baral K (1999). Transformation of phosphorus during vermicomposting. Bioresour Technol.

[CR105_31] Gliotti C, Giusquiani PL, Businelli D, Machioni A (1997). Composition changes of dissolved organic matter in a soil amended with municipal waste compost. Soil Sci.

[CR106_31] Goenadi DH, Sudharama IM (1995). Shoot initiation by humic acids of selected tropical crops grown in tissue culture. Plant Cell Rep.

[CR107_31] Goldstein J (1998). Compost suppresses diseases in the lab and fields. BioCycle.

[CR108_31] Gopal M, Gupta A, Sunil E, Thomas VG (2009). Amplification of plant beneficial microbial communities during conversion of coconut leaf substrate to vermicompost by Eudrilus sp. Curr Microbiol.

[CR109_31] Graff O, Makeschin F (1980). Beeinlussung des Ertrags von Weidelgrass (Lolium muttiflorum) Ausscheidungen von Regenwurmen dreier verschiedener Arten. Pedobiologia.

[CR110_31] Grappelli A, Galli E, Tomati U (1987). Earthworm casting effect on Agaricus bisporus fructification. Agrochimica.

[CR111_31] Guerrero RD, Guerrero LA (2006). Response of eggplant (Solanum melongena) grown in plastic containers to vermicompost and chemical fertilizer. Asia Life Sciences.

[CR112_31] Gunadi B, Blount C, Edward CA (2002). The growth and fecundity of Eisenia foetida (Savigny) in cattle solids pre-composted for different periods. Pedobiologia.

[CR113_31] Gunathilagraj K, Ravignanam T (1996). Vermicomposting of sericulture wastes. Madras Agric J.

[CR114_31] Gupta AK, Pankaj PK, Upadhyava V (2008). Effect of vermicompost, farm yard manure, biofertilizer and chemical fertilizers (N, P, K) on growth, yield and quality of lady’s finger (Abelmoschus esculentus). Pollution Research.

[CR115_31] Hahn H, Bopp M (1968). A cytokinin test with high specificity. Planta.

[CR116_31] Han J, Sun L, Dong X, Cai Z, Yang H, Wang Y, Song W (2005). Characterization of a novel plant growth-promoting bacteria strain Delftia tsuruhatensis HR4 both as a diazotroph and a potential biocontrol agent against various pathogens. Syst Appl Microbiol.

[CR117_31] Hand P, Hayes WA, Frankland JC, Satchell JE (1988). Vermicomposting of cow slurry. Pedobiologia.

[CR118_31] Haritha Devi S, Vijayalakshmi K, Pavana Jyotsna K, Shaheen SK, Jyothi K, Surekha Rani M (2009). Comparative assessment in enzyme activities and microbial populations during normal and vermicomposting. J Environ Biol.

[CR119_31] Hartenstein R, Neuhauser EF, Kaplan DL (1979). Reproductive potential of the earthworm Eisenia foetida. Oecologia.

[CR120_31] Hartenstein R, Satchell JE (1983). Assimilation by earthworm Eisenia fetida. Earthworm ecology. From Darwin to vermiculture.

[CR121_31] Hashemimajd K, Kalbasi M, Golchin A, Shariatmadari H (2004). Comparison of vermicompost and composts as potting media for growth of tomatoes. J Plant Nutr.

[CR122_31] Hassen A, Belguith K, Jedidi N, Cherif A, Cherif M, Boudabous A (2001). Microbial characterization during composting of municipal solid waste. Bioresour Technol.

[CR123_31] Hawida S, Kapari L, Ossipov V, Ramtala MJ, Ruuhola T, Haukioja E (2007). Foliar phenolics are differently associated with Epirrita autmnata growth and immuno competence. J Chem Ecol.

[CR124_31] Herms DA (2002). Effects of fertilization on insect resistance of woody ornamental plants. Environ Entomol.

[CR125_31] Hoitink HA, Kuter GA, Chen Y, Avnimelech Y (1986). Efects of composts in growth media on soil-bome pathogens. The role of organic matter in modern agriculture.

[CR126_31] Hoitink HA, Stone AG, Han DY (1997). Suppression of plant diseases by compost. Hort Sci.

[CR127_31] Hoitink HA, Grebus ME, Hayes MHB, Wilson WS (1997). Composts and Control of Plant Diseases. Humic Substances Peats and Sludges Health and Environmental Aspects.

[CR128_31] Holtzclaw KM, Sposito G (1979). Analytical properties of the soluble metal-complexing fractions in sludge-soil mixtures. IV. Determination of carboxyl groups in fulvic acid. Soil Sci Soc Am J.

[CR129_31] Huelsman MF, Edwards CA, Lawrence JL, Clarke-Harris DO (2000). A study of the effect of soil nitrogen levels on the incidence of insect pests and predators in Jamaican sweet potato (Ipomoea batatus) and Callaloo (Amaranthus). Proc Brighton Pest Control Conference: Pests and Diseases.

[CR130_31] Inbal E, Feldman M (1982). The response of a hormonal mutant of common wheat to bacteria of the Azospirillium. Israel J Bot.

[CR131_31] Ismail SA, Thampan PK (1995). Earthworms in soil fertility management. Organic Agriculture.

[CR132_31] Ismail SA (1997). Vermicology: The biology of Earthworms.

[CR133_31] Jadhav AD, Talashilkar SC, Pawar AG (1997). Influence of the conjunctive use of FYM, vermicompost andurea on growth and nutrient uptake in rice. J Maharashtra Agric Univ.

[CR134_31] Jagnow G (1987). Inoculation of cereal crops and forage grasses with nitrogen-fixing rhizosphere bacteria: a possible cause of success and failure with regard to yield response - a review. Z Pflanzenernaehr Dueng Bodenkde.

[CR135_31] Jambhekar H (1992). Use of earthworm as a potential source of decompose organic wastes.

[CR136_31] Jannsson RK, Smilowitz Z (1986). Influence of nitrogen on population parameters of potato insects: abundance, population growth and within-plant distribution of the green peach aphid, Myzus persicae (Homoptera: Aphididae). Environ Entomol.

[CR137_31] Jha BK, Gandhi Pragash M, Cletus J, Raman G, Sakthivel N (2009). Simultaneous phosphate solubilization potential and antifungal activity of new fluorescent pseudomonad strains, Pseudomonas aeruginosa, P. plecoglossicida and P. mosselii. W J Microbiol Biotech.

[CR138_31] Jolly JM, Lappin-Scott HM, Anderson JM, Clegg CD (1993). Scanning electron microscopy of the gut microflora of two earthworms: Lumbricus terrestris and Octolasion cyaneum. Microbial Ecol.

[CR139_31] Kale RD, Bano K, Dass MC, Senapati BK, Mishra PC (1986). Field Trials with vermicompost (vee comp. E. 8. UAS) on organic fertilizers. Proceedings of the national seminar on organic waste utilization.

[CR140_31] Kale RD, Bano K (1988). Earthworm cultivation and culturing techniques for the production of vee COMP83E UAS. Mysore J Agric Sci.

[CR141_31] Kale RD, Mallesh BC, Bano K, Bagyaray DJ (1992). Influence of vermicompost application on the available macronutrients and selected microbial populations in paddy field. Soil Biol Biochem.

[CR142_31] Kale RD (1995). Vermicomposting has a bright scope. Indian Silk.

[CR143_31] Kalembasa D (1996). The influence of vermicomposts on yield and chemical composition of tomato. Zesz Probl Post Nauk Roln.

[CR144_31] Kannangowa T, Utkhede RS, Paul JW, Punja ZK (2000). Effect of mesophilic and thermophilic composts on suppression of Fusarium root and stem rot of greenhouse cucumber. Canad J Microbiol.

[CR145_31] Karmegam N, Alagermalai K, Daniel T (1999). Effect of vermicompost on the growth and yield of greengram (Phaseolus aureus Rob.). Trop Agric.

[CR146_31] Karmegam N, Daniel T (2000). Effect of biodigested slurry and vermicompost on the growth and yield of cowpea (Vigna unguiculata (L.). Environ Ecol.

[CR147_31] Karmegam N, Daniel T (2008). Effect of vermi-compost and chemical fertilizer on growth and yield of Hyacinth Bean (Lablab purpureas). Dynamic Soil, Dynamic Plant, Global Science Books.

[CR148_31] Karsten GR, Drake HL (1995). Comparative assessment of the aerobic and anaerobic microfloras of earthworm guts and forest soils. Appl Environ Microbiol.

[CR149_31] Karsten GR, Drake HL (1997). Denitrifying bacteria in the earthworm gastrointestinal tract and in vivo emission of nitrous oxide (N2O) by earthworms. Appl Environ Microbiol.

[CR150_31] Kerry B, Edwards CA, Stinner BR, Stinner D, Rabatin S (1988). Fungal parasites of cyst nematodes. Biological Interactions in Soil.

[CR151_31] Khambata SR, Bhat JV (1953). Studies on a new oxalate-decomposing bacterium, Pseudomonas oxalaticus. J Bacteriol.

[CR152_31] Knuutinen J, Palm H, Hakala H, Haimi J, Huhta V, Salminen J (1990). Polychlorinated phenols and their metabolites in soil and earthworms of a saw mill environment. Chemosphere.

[CR153_31] Kolodziej M, Kostecka J (1994). Some qualitative features of the cucumbers and carrots cultivated on the vermicompost. Zeszyty Naukowe Akademii Rolniczej W Krakowie.

[CR154_31] Kooch Y, Jalilvand H (2008). Earthworm as ecosystem engineers and the most important detritivors in forst soils. Pak J Boil Sci.

[CR155_31] Kostecka J, Blazej JB, Kolodziej M (1996a). Investigations on application of vermicompost in potatoes farming in second year of experiment. Zeszyty Naukowe Akademii Rolniczej W Krakowie.

[CR156_31] Koul O (2008). Phytochemicals and insect control: an antifeedant approach. Crit Rev Plant Sci.

[CR157_31] Krishnamoorthy RV, Vajranabhiah SN (1986). Biological activity of earthworm casts: An assessment of plant growth promoter levels in casts. Proc Indian Acad Sci (Anim Sci).

[CR158_31] Kristufek V, Ravasz K, Pizl V (1993). Actinomycete communities in earthworm guts and surrounding soil. Pedobiologia.

[CR159_31] Kurowska A, Gora J, Kalemba D (1990). Effects of plant phenols on insects. Pol Wiad Chem.

[CR160_31] Kuter GA, Nelson GB, Hoitink HA, Madden LV (1983). Fungal population in container media amended with composted hardwood bark suppressive and conductive to Rhizoctonia damping-off. Phytopathology.

[CR161_31] Lavelle E, Barois I, Martin A, Zaidi Z, Schaefer R, Clarholm M, Bergstrom L (1989). Management of earthworm populations in agro-ecosystems: A possible way to maintain soil quality?. Ecology of Arable Land: Perspectives and Challenges.

[CR162_31] Lavelle P, Martin A (1992). Small-scale and large-scale effects of endogeic earthworms on soil organic matter dynamics in soils of the humid tropics. Soil Biol Biochem.

[CR163_31] Lazarovits G, Tenuta M, Conn KL, Gullino ML, Katan J, Matta A (2000). Utilization of high nitrogen and swine manure amendments for control of soil-bome diseases: efficacy and mode of action. Acta Hortic.

[CR164_31] Lazcano C, Gomez-Brandon M, Dominguez J (2008). Comparison of the effectiveness of composting and vermicomposting for the biological stabilization of cattle manure. Chemosphere.

[CR165_31] Lee KE (1985). Earthworms: Their Ecology and Relationships with Soils and Land Use.

[CR166_31] Lee YS, Bartlett RJ (1976). Stimulation of plant growth by humic substances. Soil Sci Soc Am J.

[CR167_31] Lunt HA, Jacobson HGM (1944). The chemical composition of earthworm casts. Soil Sci.

[CR168_31] Maboeta MS, Van Rensburg L (2003). Vermicomposting of industrially produced wood chips and sewage sludge utilizing Eisenia foetida. Ecotoxicol Environ Saf.

[CR169_31] Madsen EL, Alexander M (1982). Transport of Rhizobium and Pseudomonas through soil. Soil Sci Soc Am J.

[CR170_31] Mahanil S, Attajarusit J, Stout MJ, Thipayong P (2008). Over expression of tomato phenol oxidase increases resistance to the common cutworm. Plant Sci.

[CR171_31] Maheswarappa HP, Nanjappa HV, Hegde MR (1999). Influence of organic manures on yield of arrowroot, soil physico-chemical and biological properties when grown as intercrop in coconut garden. Ann Agr Res.

[CR172_31] Mahmoud SA, Ramadan Z, Thabet EM, Khater T (1984). Production of plant growth promoting substance rhizosphere organisms. Zentrbl Mikrobiol.

[CR173_31] Makulec G (2002). The role of Lumbricus rubellus Hoffm. In determining biotic and abiotic properties of peat soils. Pol J Ecol.

[CR174_31] Marinari S, Masciandaro G, Ceccanti B, Grego S (2000). Influence of organic and mineral fertilisers on soil biological and physical properties. Bioresour Technol.

[CR175_31] Martin JP (1976). Darwin on earthworms: the formation of vegetable moulds.

[CR176_31] Masciandaro G, Ceccanti B, Gracia C (1997). Soil agro-ecological management: fertigation and vermicompost treatments. Bioresour Technol.

[CR177_31] Masciandaro G, Ceccanti B, Gracia C (2000). `In situ' vermicomposting of biological sludges and impacts on soil quality. Soil Biol Biochem.

[CR178_31] Meena RN, Singh Y, Singh SP, Singh JP, Singh K (2007). Effect of sources and level of organic manure on yield, quality and economics of garden pea (Pisum sativam L.) in eastern uttar pradesh. Vegetable Science.

[CR179_31] Mitchell A, Edwards CA (1997). The production of vermicompost using Eisenia fetida from cattle manure. Soil Biol Biochem.

[CR180_31] Mitchell A (1997). Production of Eisenia fetida and vermicompost from feed-lot cattle manure. Soil Biol Biochem.

[CR181_31] Mitchell MJ, Hornor SG, Abrams BI (1980). Decomposition of sewage sludge in drying beds and the potential role of the earthworm, Eisenia foetida. J Environ Qual.

[CR182_31] Mitchell MJ, Hartenstein R (1978). Role of invertebrates and microorganisms in sludge deomposition. Utilisation of soil organisms in sludge management. natural technology information services.

[CR183_31] Monroy F, Aira M, Domínguez J (2009). Reduction of total coliform numbers during vermicomposting is caused by short-term direct effects of earthworms on microorganisms and depends on the dose of application of pig slurry. Sci Tot Environ.

[CR184_31] Moody SA, Piearce TG, Dighton J (1996). Fate of some fungal spores associated with wheat straw decomposition on passage through the guts of Lumbricus terrestris and Aporrectodea longa. Soil Biol Biochem.

[CR185_31] Morra L, Palumbo AD, Bilotto M, Ovieno P, Ptcascia S (1998). Soil solarization: organic fertilization grafting contribute to build an integrated production system in a tomato-zucchini sequence. Colture-Protte.

[CR186_31] Munnoli PM, Da Silva JAT, Saroj B (2010). Dynamics of the soil-earthworm-plant relationship: a review. Dynamic soil, dynamic plant.

[CR187_31] Muscolo A, Bovalo F, Gionfriddo F, Nardi S (1999). Earthworm humic matter produces auxin-like effect on Daucus carota cell growth and nitrate metabolism. Soil Biol Biochem.

[CR188_31] Muscolo A, Felici M, Concheri G, Nardi S (1993). Effect of earthworm humic substances on esterase and peroxidase activity during growth of leaf explants of Nicotiana plumbaginifolia. Biol Fertil Soils.

[CR189_31] Muscolo A, Panuccio MR, Abenavoli MR, Concheri G, Nardi S (1996). Effect of molecular complexity acidity of earthworm faeces humic fractions on glutamale dehydrogenase, glutamine synthetase, and phosphoenolpyruvate carboxylase in Daucus carota II cells. Biol Fertil Soils.

[CR190_31] Mylonas VA, Mccants CB (1980). Effects of humic and fulvic acids on growth of tobacco. I. Root initiation and elongation. Plant Soil.

[CR191_31] Nagavallemma KP, Wani SP, Stephane L, Padmaja VV, Vineela C, Babu Rao M, Sahrawat KL (2004). Vermicomposting: Recycling wastes into valuable organic fertilizer. Global Theme on Agrecosystems Report no.8. Patancheru 502324.

[CR192_31] Nakamura Y (1996). Interactions between earthworms and microorganisms in biological control of plant root pathogens. Farming Jpn.

[CR193_31] Nakasone AK, Bettiol W, de Souza RM (1999). The effect of water extracts of organic matter on plant pathogens. Summa Phytopathology.

[CR194_31] Nardi S, Arnoldi G, Dell'Agnola G (1988). Release of hormone-like activities from Alloborophora rosea and Alloborophora caliginosa feces. J Soil Sci.

[CR195_31] Nardi S, Dell'Agnola G, Nuti PM (1983). Humus production from farmyard wastes by vermicomposting. Proc. Int. Symp. On Agricultural and Environmental Prospects in Earthworm Farming.

[CR196_31] Ndegwa PM, Thompson SA, Das KC (2000). Effects of stocking density and feeding rate on vermicomposting of biosolids. Bioresour Technol.

[CR197_31] Nechitaylo TY, Yakimov MM, Godinho M, Timmis KN, Belogolova E, Byzov BA (2010). Effect of the earthworms Lumbricus terrestris and Aporrectodea caliginosa on bacterial diversity in soil. Microbial Ecol.

[CR198_31] Nethra NN, Jayaprasad KV, Kale RD (1999). China aster (Callistephus chinensis (L)) cultivation using vermicompost as organic amendment. Crop Research, Hisar.

[CR199_31] Nielson RL (1965). Presence of plant growth substances in earthworms demonstrated by paper chromatography and the Went pea test. Nature.

[CR200_31] Orozco FH, Cegarra J, Trujillo LM, Roig A (1996). Vermicomposting of coffee pulp using the earthworm Eisenia fetida: effects on C and N contents and the availability of nutrients. Biol Fertil Soils.

[CR201_31] Park SR, Cho EJ, Yu KH, Kim YS, Suh JJ, Chang CS (1996). Endogenous phenoloxidase from an earthworm Lumbricus rubellus. Tongmul Hakoehi.

[CR202_31] Parle JN (1963). A Microbiological Study of Earthworm Casts. J Gen Microbiol.

[CR203_31] Parthasarathi K, Ranganathan LS (1998). Pressmud vermicast are hot spots of fungi and bacteria. Ecol Environ Cons.

[CR204_31] Pathma J, Ayyadurai N, Sakthivel N (2010). Assessment of Genetic and Functional Relationship of Antagonistic Fluorescent Pseudomonads of Rice Rhizosphere by Repetitive Sequence, Protein Coding Sequence and Functional Gene Analyses. J Microbiol.

[CR205_31] Pathma J, Kamaraj Kennedy R, Sakthivel N, Maheswari DK (2011a). Mechanisms of fluorescent pseudomonads that mediate biological control of phytopathogens and plant growth promotion of crop plants. Bacteria in Agrobiology: Plant Growth Responses.

[CR206_31] Pathma J, Rahul GR, Kamaraj Kennedy R, Subashri R, Sakthivel N (2011). Secondary metabolite production by bacterial antagonists. Journal of Biological Control.

[CR207_31] Patil SL, Sheelavantar MN (2000). Effect of moisture conservation practices, organic sources and nitrogen levels on yield, water use and root development of rabi sorghum (Sorghum bicolor (L.)) in the vertisols of semiarid tropics. Ann Agric Res.

[CR208_31] Patriquin DG, Baines D, Abboud A, Cook HF, Lee HC (1995). Diseases, pests and soil fertility. Soil Management in Sustainable Agriculture.

[CR209_31] Pedersen JC, Hendriksen NB (1993). Effect of passage through the intestinal tract of detritivore earthworms (Lumbricus spp.) on the number of selected gram-negative and total bacteria. Biol Fertil Soils.

[CR210_31] Petersen H, Luxton MA (1982). A comparative analysis of soil fauna populations and their role in decomposition process. Oikos.

[CR211_31] Phelan PL, Norris KH, Mason JF (1996). Soil management history and host preference by Ostrinia nubilatis: evidence for plant mineral balance mediating insect-plant interactions. Environ Entom.

[CR212_31] Phelan PL, Magadoff F, Well RR (2004). Connecting below-ground and above-ground food webs: the role of organic matter in biological buffering. Soil Organic Matter in sustainable agriculture.

[CR213_31] Pinel N, Davidson SK, Stahl DA (2008). Verminephrobacter eiseniae gen. nov., sp. nov., a nephridial symbiont of the earthworm Eisenia foetida (Savigny). Int J Syst Evol Microbiol.

[CR214_31] Pitt D, Tilston EL, Groenhof AC, Szmidt RA (1998). Recycled organic materials (ROM) in the control of plant disease. Acta Hortic.

[CR215_31] Pizl V, Novokova A (1993). Interactions between microfungi and Eisenia andrei (Oligochaeta) during cattle manure vermicomposting. Pedobiologia.

[CR216_31] QiTian S (2004). Research on prevention and elimination of agricultural pests by ginkgo phenols phenolic acids. Chem Ind For Prod.

[CR217_31] Raguchander T, Rajappan K, Samiyappan R (1998). Influence of biocontrol agents and organic amendments on soybean root rot. Int J Trop Agri.

[CR218_31] Ramesh P (2000). Effects of vermicomposts and vermicornposting on damage by sucking pests to ground nut (Arachis hypogea). Indian J Agri Sci.

[CR219_31] Rao KR, Rao PA, Rao KT (2001). Influence of fertilizers and manures on the population of coccinellid beetles and spiders in groundnut ecosystem. Ann Plant Protect Sci.

[CR220_31] Rao KR (2002). Induce host plant resistance in the management sucking pests of groundnut. Ann Plant Protect Sci.

[CR221_31] Rao KR (2003). Influence of host plant nutrition on the incidence of Spodoptera litura and Helicoverpa armigera on groundnuts. Indian J Entomol.

[CR222_31] Ravindra NP, Raman G, Badri Narayanan K, Sakthivel N (2008). Assessment of genetic and functional diversity of phosphate solubilizing fluorescent pseudomonads isolated from rhizospheric soil. BMC Microbiol.

[CR223_31] Reeh U (1992). Influence of population densities on growth and reproduction of the earthworm Eisenia andrei on pig manure. Soil Biol Biochem.

[CR224_31] Ribeiro CF, Mizobutsi EH, Silva DG, Pereira JCR, Zambolim L (1998). Control of Meloidognye javanica on lettuce with organic amendments. Fitopatol Brasileira.

[CR225_31] Riffaldi R, Levi-Minzi R (1983). Osservazioni preliminari sul ruolo dell Eisenia foetida nell'umificazione del letame. Agrochimica.

[CR226_31] Rivera AMC, Wright ER, López MV, Fabrizio MC (2004). Temperature and dosage dependent suppression of damping-off caused by Rhizoctonia solani in vermicompost amended nurseries of white pumpkin. Phyton.

[CR227_31] Rodriguez JA, Zavaleta E, Sanchez P, Gonzalez H (2000). The effect of vermicomposts on plant nutrition, yield and incidence of root and crown rot of gerbera (Gerbera jamesonii H. Bolus). Fitopatol.

[CR228_31] Rodriguez-Kabana R (1986). Organic and inorganic amendments to soil as nematode suppressants. J Nematol.

[CR229_31] Rouelle J, Satchell JE (1983). Introduction of an amoeba and Rhizobium Japonicum into the gut of Eisenia fetida (Sav.) and Lumbricus terrestris L. Earthworm Ecology: From Darwin to Vermiculture.

[CR230_31] Sánchez-Monedero MA, Roig A, Paredes C, Bernal MP (2001). Nitrogen transformation during organic waste composting by the Rutgers system and its effects on ph, EC and maturity of the composting mixtures. Bioresour Technol.

[CR231_31] Sainz MJ, Taboada-Castro MT, Vilariño A (1998). Growth, mineral nutrition and mycorrhizal colonization of red clover and cucumber plants grown in a soil amended with composted urban wastes. Plant Soil.

[CR232_31] Saumaya G, Giraddi RS, Patil RH (2007). Utility of vermiwash for the management of thrips and mites on chilli (Capiscum annum) amended with soil organics. Karnataka J Agric Sci.

[CR233_31] Scheu S (1992). Automated measurement of the respiratory response of soil micro-compartments: active microbial biomass in earthworm faeces. Soil Biol Biochem.

[CR234_31] Scheuerell SJ, Sullivan DM, Mahaffee WF (2005). Suppression of seedling damping-off caused by Pythium ultimum, and Rhizoctonia solani in container media amended with a diverse range of Pacific Northwest compost sources. Phytopathology.

[CR235_31] Schmidt O, Doubre BM, Ryder MH, Killman K (1997). Population dynamics of Pseudomonas corrugata 2140R LUX8 in earthworm food and in earthworm cast. Soil Biol Biochem.

[CR236_31] Sembdner G, Borgman E, Schneider G, Liebisch HW, Miersch O, Adam G, Lischewski M, Schieber K (1976). Biological activity of some conjugated gibberellins. Planta.

[CR237_31] Senesi N, Saiz-Jimenez C, Miano TM (1992). Spectroscopic characterization of metal-humic acid-like complexes of earthworm-composted organic wastes. Sci Total Environ.

[CR238_31] Sharma S, Pradhan K, Satya S, Vasudevan P (2005). Potentiality of earthworms for waste management and in other uses - A Review. The Journal of American Science.

[CR239_31] Sharpley AN, Syers JK (1976). Potential role of earthworm casts for the phosphorous enrichment of runoff waters. Soil Biol Biochem.

[CR240_31] Shi-wei Z, Fu-zhen H, Veeresh GK, Rajagopal D, Viraktamath CA (1991). The nitrogen uptake efficiency from 15N labeled chemical fertilizer in the presence of earthworm manure (cast). Advances in Management and Conservation of Soil Fauna.

[CR241_31] Siddiqui ZA, Mahmood I (1999). Role of bacteria in the management of plant parasitic nematodes: a review. Bioresour Technol.

[CR242_31] Sidhu J, Gibbs RA, Ho GE, Unkovich I (2001). The role of indigenous microorganisms in suppression of Salmonella regrowth in composted biosolids. Water Res.

[CR243_31] Simsek Ersahin Y, Haktanir K, Yanar Y (2009). Vermicompost suppresses Rhizoctonia solani Kühn in cucumber seedlings. J Plant Dis Protect.

[CR244_31] Singh R, Sharma RR, Kumar S, Gupta RK, Patil RT (2008). Vermicompost substitution influences growth, physiological disorders, fruit yield and quality of strawberry (Fragaria x ananassa Duch.). Bioresour Technol.

[CR245_31] Singh UP, Maurya S, Singh DP (2003). Antifungal activity and induced resistance in pea by aqueous extract of vermicompost and for control of powdery mildew of pea and balsam. J Plant Dis Protect.

[CR246_31] Singleton DR, Hendrixb PF, Colemanb DC, Whitmana WB (2003). Identification of uncultured bacteria tightly associated with the intestine of the earthworm Lumbricus rubellus (Lumbricidae; Oligochaeta). Soil Biol Biochem.

[CR247_31] Sinha RK, Agarwal S, Chauhan K, Valani D (2010). The wonders of earthworms and its vermicompost in farm production: Charles Darwin’s ‘friends of farmers’, with potential to replace destructive chemical fertilizers from agriculture. Agricultural sciences.

[CR248_31] Sinha RK, Bharambe G, Chaudhari U (2008). Sewage treatment by vermifiltration with synchronous treatment of sludge by earthworms: a low-cost sustainable technology over conventional systems with potential for decentralization. The Environmentalist.

[CR249_31] Sinha RK, Heart S, Agarwal S, Asadi R, Carretero E (2002). Vermiculture technology for environmental management: study of the action of the earthworms Eisenia foetida, Eudrilus euginae and Perionyx excavatus on biodegradation of some community wastes in India and Australia. The Environmentalist.

[CR250_31] Sinha RK, Herat S, Valani D, Chauhan K (2009). Vermiculture and sustainable agriculture. Am-Euras J Agric and Environ Sci, IDOSI Publication.

[CR251_31] Sipes BS, Arakaki AS, Schmitt DP, Hamasaki RT (1999). Root-knot nematode management in tropical cropping systems with organic products. J Sustain Agr.

[CR252_31] Sreenivas C, Muralidhar S, Rao MS (2000). Vermicompost, a viable component of IPNSS in nitrogen nutrition of ridge gourd. Ann Agr Res.

[CR253_31] Steffen KL, Dan MS, Harper JK, Fleischer SJ, Mkhize SS, Grenoble DW, MacNab AA, Fager K (1995). Evaluation of the initial season for implementation of four tomato production systems. J Am Soc Hort Sci.

[CR254_31] Stephens PM, Davoren CW, Doube BM, Ryder MH (1993). Reduced superiority of Rhizoctonia solani disease on wheat seedlings associated with the presence of the earthworm Aporrectodea trapezoids. Soil Biol Biochem.

[CR255_31] Stephens PM, Davoren CW, Ryder MH, Doube BM, Correll RL (1994). Field evidence for reduced severity of Rhizoctonia bare-patch disease of wheat, due to the presence of the earthworms Aporrectodea rosea and Aporrectodea trapezoides. Soil Biol Biochem.

[CR256_31] Stephens PM, Davoren CW, Ryder MH, Doube BM (1994). Influence of the earthworm Aporrectodea trapezoides (Lumbricidae) on the colonization of alfalfa (Medicago sativa L.) roots by Rhizobium melilotti strain LS-30R and the survival of L5-30R in soil. Biol Fertil Soils.

[CR257_31] Stephens PM, Davoren CW (1997). Influence of the earthworms Aporrectodea trapezoides and A. rosea on the disease severity of Rhizoctonia solani on subterranean cloves and ryegrass. Soil Biol Biochem.

[CR258_31] Stone AG, Scheurell SJ, Darby HM, Magdoff F, Weil (2004). Suppression of soilborne diseases in field agricultural systems: organic matter management, cover cropping and other cultural practices. Soil Organic Matter in Sustainable Agriculture.

[CR259_31] Subler S, Edwards CA, Metzger PJ (1998). Comparing vermicomposts and composts. Biocycle.

[CR260_31] Sudhakar K, Punnaiah KC, Krishnayya PV (1998). Influence of organic and inorganic fertilizers and certain insecticides on the incidence of shoot and fruit borer, Leucinodes orbonalis Guen, infesting brinjal. J Entomol Res.

[CR261_31] Suhane RK (2007). Vermicompost.

[CR262_31] Summers G, Felton GW (1994). Prooxidation effects of phenolic acids on the generalist herbivore Helicoverpa zea: potential mode of action of phenolic compounds on plant anti-herbivory chemistry. Insect Biochem Mol Biol.

[CR263_31] Sunish KR, Ayyadurai N, Pandiaraja P, Reddy AV, Venkateshwarlu Y, Prakash O, Sakthivel N (2005). Characterization of antifungal metabolite produced by a new strain Pseudomonas aeruginosa PuPa3 that exhibits broad-spectrum antifungal activity and biofertility traits. J Appl Microbiol.

[CR264_31] Suthar S (2010). Evidence of plant hormone like sub-stances in vermiwash: An ecologically safe option of synthetic chemicals for sustainable farming. J Ecol Eng.

[CR265_31] Suthar S, Singh S (2008). Vermicomposting of domestic waste by using two epigeic earthworms (Perionyx excavatus and Perionyx sansibaricus). Int J Evniron Sci and Technol.

[CR266_31] Swathi P, Rao KT, Rao PA (1998). Studies on control of root-knot nematode Meloidogyne incognita in tobacco miniseries. Tobacco Res.

[CR267_31] Szcech M, Rondomanski W, Brzeski MW, Smolinska U, Kotowski JF (1993). Suppressive effect of a commercial earthworm compost on some root infecting pathogens of cabbage and tomato. Biol Agric and Hortic.

[CR268_31] Szczech M, Smolinska U (2001). Comparison of suppressiveness of vermicomposts produced from animal manures and sewage sludge against Phytophthora nicotianae Breda de Haan var. nicotiannae. J Phytopathology.

[CR269_31] Szczech MM (1999). Suppressiveness of vermicomposts against fusarium wilt of tomato. J Phytopathology.

[CR270_31] Tajbakhsh J, Abdoli MA, Mohammadi Goltapeh E, Alahdadi I, Malakouti MJ (2008). Trend of physico-chemical properties change in recycling spent mushroom compost through vermicomposting by epigeic earthworms Eisenia foetida and E. andrei. J Agric Technol.

[CR271_31] Tan KH, Tantiwiramanond D (1983). Effect of humic acids on nodulation and dry matter production of soybean, peanut, and clover. Soil Sci Soc Am J.

[CR272_31] Thoden TC, Korthals GW, Termorshuizen (2011). Organic amendments and their influences on plant-parasitic and free living nematodes: a promosing method for nematode management. Nematology.

[CR273_31] Tiquia SM (2005). Microbiological parameters as indicators of compost maturity. J Appl Microbiol.

[CR274_31] Tiunov AV, Scheu S (2000). Microfungal communities in soil litter and casts of Lumbricus terrestris (Lumbricidae): a laboratory experiment. Appl Soil Ecol.

[CR275_31] Tiwari SC, Tiwari BK, Mishra RR (1989). Microbial populations, enzyme activities and nitrogen, phosphorous, potassium enrichment in earthworm casts and in the surrounding soil of pine apple plantation. Biol Fertil Soils.

[CR276_31] Tomati U, Grapppelli A, Galli E, Bonvicini Paglioi AM, Omodeo P (1987). The presence of growth regulators in earthworm worked waste. On Earthworms. Proceedings of International Symposium on Earthworms, Selected Symposia and Monographs, Union Zoologica Italian, 2.

[CR277_31] Tomati U, Grapppelli A, Galli E (1988). The hormone-like effect of earthworm casts on plant growth. Biol Fertil Soils.

[CR278_31] Toyota K, Kimura M (2000). Microbial community indigenous to the earthworm Eisenia foetida. Biol Fertil Soils.

[CR279_31] Trevors JT (1984). Dehydrogenase activity in soil. A comparison between the INT and TTC assay. Soil Biol Biochem.

[CR280_31] Umesh B, Mathur LK, Verma JN, Srivastava (2006). Effects of vermicomposting on microbiological flora of infected biomedical waste. ISHWM Journal.

[CR281_31] Vadiraj BA, Siddagangaiah D, Potty SN (1998). Response of coriander (Coriandrum sativum L.) cultivars to graded levels of vermicompost. J Spices Aromatic Crops.

[CR282_31] Valdrighi MM, Pera A, Agnolucci M, Frassinetti S, Lunardi D, Vallini G (1996). Effects of compost-derived humic acids on vegetable biomass production and microbial growth within a plant (Cichorium intybus) soil system: a comparative study. Agric Ecosyst Environ.

[CR283_31] Valenzuela O, Gluadia Y, Gallardo S (1997). Use of vermicompost as a growing medium for tomato seedlings (cv. Pltense). Revista Cientifica Agropecuaria.

[CR284_31] Vaz-Moreira I, Maria E, Silva CM, Manaia Olga C, Nunes (2008). Diversity of Bacterial Isolates from Commercial and Homemade Composts. Microbial Ecol.

[CR285_31] Vinken R, Schaeffer A, Ji R (2005). Abiotic association of soil-borne monomeric phenols with humic acids. Org Geochem.

[CR286_31] Vivas A, Moreno B, Garcia-Rodriguez S, Benitez E (2009). Assessing the impact of composting and vermicomposting on bacterial community size and structure, and functional diversity of an olive-mill waste. Bioresour Technol.

[CR287_31] Webster KA (2005). Vermicompost increases yield of cherries for three years after a single application.

[CR288_31] Weltzien HC (1989). Some effects of composted organic materials on plant health. Agric Ecosyst Environ.

[CR289_31] Wilson DP, Carlile WR (1989). Plant growth in potting media containing worm-worked duck waste. Acta Hortic.

[CR290_31] Yardim EN, Arancon NQ, Edwards CA, OliverTJ BRJ (2006). Suppression of tomato hornworm (Manduca quinquemaculata) and cucumber beetles (Acalymma vittatum and Diabotrica undecimpunctata) populations and damage by vermicomposts. Pedobiologia.

[CR291_31] Yardim EN, Edwards CA (2003). Effects of organic and synthetic fertilizer sources on pest and predatory insects associated with tomatoes. Phytoparasitica.

[CR292_31] Yasir M, Aslam Z, Kim SW, Lee SW, Jeon CO, Chung YR (2009). Bacterial community composition and chitinase gene diversity of vermicompost with antifungal activity. Bioresour Technol.

[CR293_31] Yasir M, Aslam Z, Song GC, Jeon CO, Chung YR (2009b). Eiseniicola composti gen. nov., sp. nov., with antifungal activity against plant pathogenic fungi. Int J Sys Evol Microbiol.

[CR294_31] Yeates GW (1981). Soil nematode populations depressed in the presence of earthworms. Pedobiologiaogia.

[CR295_31] Zhang BG, Li GT, Shen TS, Wang JK, Sun Z (2000). Changes in microbial biomass C, N, and P and enzyme activities in soil incubated with the earthworms Metaphire guillelmi or Eisenia foetida. Soil Biol Biochem.

